# Correlation between Ferroptosis-Related Gene Signature and Immune Landscape, Prognosis in Breast Cancer

**DOI:** 10.1155/2022/6871518

**Published:** 2022-10-11

**Authors:** Jiahao Zhu, Qingqing Chen, Ke Gu, You Meng, Shengjun Ji, Yutian Zhao, Bo Yang

**Affiliations:** ^1^Department of Radiotherapy and Oncology, The Affiliated Hospital of Jiangnan University, Wuxi, Jiangsu 214000, China; ^2^Department of Radiotherapy and Oncology, The Affiliated Suzhou Hospital of Nanjing Medical University, Gusu School, Nanjing Medical University, Suzhou, Jiangsu 215000, China; ^3^Department of Breast Surgery, The Affiliated Suzhou Hospital of Nanjing Medical University, Gusu School, Nanjing Medical University, Suzhou, Jiangsu 215000, China

## Abstract

Breast cancer (BC) is the most commonly diagnosed cancer and second leading cause of cancer-related death in women worldwide. Ferroptosis, an iron-dependent newly discovered mode of cell death, can be induced by lenaltinib and plays an important role in the biological behaviors of BC. Therefore, the prognostic value of ferroptosis-related genes (FRGs) in BC warrants further investigation. FRG expression profiles and clinical data were downloaded from The Cancer Genome Atlas (TCGA) database and Gene Expression Omnibus (GEO). Immune-related pathways were found in the functional analysis. Significant differences in enrichment scores for immune cells were observed. Some patients from TCGA-BRCA were included as the training cohort. A six-gene prediction signature was constructed with the least absolute shrinkage and selection operator Cox regression. This model was validated in the rest of the TCGA-BRCA and GEO cohort. The expressions of the six FRGs were verified with real-time quantitative polymerase chain reaction and immunohistochemistry in the Human Protein Atlas. Relapse or metastasis was more likely in the high-risk group. Risk score was an independent predictor of disease-free survival. Collectively, the ferroptosis-related risk model established in this study may serve as an effective tool to predict the prognosis in BC.

## 1. Introduction

Breast cancer (BC) ranks first in terms of incidence among newly diagnosed malignancies and is the main leading cause of tumor-related death worldwide in women [1]. BC is a highly heterogeneous disease and could be divided into four subtypes, namely, luminal A, luminal B, Her-2 overexpression, and triple negative, which account for 50%, 14.1%, 12.7%, and 23.2% of all BC cases, respectively [2]. Currently, surgery, radiotherapy, chemotherapy, endocrinotherapy, targeted therapy, and even immunotherapy have been applied for the treatment of BC, with favorable outcomes in the last decades. However, the prognosis in BC is still poor especially in triple-negative or advanced BC with 5-year survival rates ranging from 23.4% to 57% [3]. Previous studies established several multigene signature models to predict the prognosis of BC. The prediction value of the 21-gene assay, which includes 16 tumor-associated genes and five reference genes, was first validated in the National Surgical Adjuvant Breast and Bowel Project (NSABP) B14 trial [4]. This study showed 10-year distant recurrence rates of 6.8%, 14.3%, and 30.5% in low-, medium-, and high-risk groups, respectively. Then, a 21-gene assay was recommended by the National Comprehensive Cancer Network to predict the risk of distant recurrence in patients with node-negative, estrogen receptor-positive BC who had been treated with tamoxifen [5]. Later, studies of Sparano further validated the production value, expanded the applicable population, and provided guidance for BC treatment [6, 7]. PAM50 signature is another multigene model that could provide risk stratification and predict prognosis in BC [8]. Its prognostic value has been validated for patients with BC in a large independent cohort with a 15-year follow-up.

Ferroptosis, first proposed in 2012, is an iron-dependent programmed cell death caused by the accumulation of lipid-based reactive oxygen species (ROS) [9, 10]. Ferroptosis is influenced by the metabolism and expression of specific genes, making ferroptosis-related genes (FRGs) an effective biomarker for predicting prognosis in various malignancies, including hepatocellular [11], colon [12], ovarian [13], esophageal [14], and renal carcinoma [15]. Previous studies showed that ferroptosis plays an important role in BC. Some genes such as *ACSL4*, *PUFAs*, and *TP53* promote the progress of ferroptosis. GPX4 is known to regulate ferroptosis negatively and lead to drug resistance in BC [16–18]. Ma et al. found that the death of BC cells induced by siramesine and lapatinib had a close association with increased Lip-ROS and FeCl3 productions, which imply that the potential treatment target related with ferroptosis must be investigated [19]. Another study demonstrated that holo-lactoferrin contributes to the progression of ferroptosis by promoting the expression of Lip-ROS in BC cells when combined with explosion of a 4 Gy electron beam, which suggests that ferroptosis further enhances the radiosensitivity of BC cells during radiation [20]. Besides growth suppression and promotion of radiosensitivity, ferroptosis plays a vital role in the metastasis of BC. A previous study demonstrated that BC cell death induced by neratinib could be reversed by administration of liproxstatin 1, a ferroptosis inhibitor rather than an apoptosis inhibitor [21]. It showed that lenaltinib significantly inhibited liver, lung, and brain metastases in Her-2 overexpression BC model nude mice.

In the present study, we first downloaded the mRNA expression profiles and corresponding clinical data of BC patients from public databases to examine the relationships between FRGs and BC recurrence or metastasis. Then, we established a prognostic multigene signature with the least absolute shrinkage and selection operator (LASSO) Cox regression in some patients in the TCGA-BRCA cohort and validated it in the rest of TCGA and GEO cohort. The expressions of six FRGs were verified with real-time quantitative polymerase chain reaction (qRT-PCR) and immunohistochemistry. Finally, risk prediction nomography and functional enrichment analysis were performed to examine the underlying mechanisms.

## 2. Materials and Methods

### 2.1. Data Collection

The RNA sequencing (RNA-seq) dataset and corresponding clinical information of BC were obtained from The Cancer Genome Atlas (TCGA; https://tcga-data.nci.nih.gov/tcga/). The expression profile of GSE21653, the validation cohort consisting of 248 BC cases, was selected from the Gene Expression Omnibus database (GEO; https://www.ncbi.nlm.nih.gov/geo/). Sixty FRGs were retrieved from previous studies [22–25].

### 2.2. BC Subclass Identification

Consensus clustering analysis was performed using FRGs. First, ferroptosis-related candidate genes significantly associated with overall survival (OS) in the TCGA-BRCA database were identified using Cox regression analysis. Then, genes with significant prognostic values (*p* < 0.05) were selected for sample clustering. Clustering methods were performed, and the best cluster number was chosen as the coexistence correlation coefficient *K* value with the “ConsensusClusterPlus” R package.

### 2.3. Differential Expression and Functional Enrichment Analyses

The “limma” R package was used to identify the differentially expressed genes (DEGs) between the different clusters with a false discovery rate (FDR) < 0.05 and logFC > |mean (abs (logFC)) + 2∗SD (abs (logFC))| in TCGA cohort. The “clusterProfiler” R package was used to conduct Gene Ontology (GO) and Kyoto Encyclopedia of Genes and Genomes (KEGG) analyses based on the DEGs. The “ESTIMATE” R package was used to calculate the StromalScore, ImmuneScore, and ESTIMATEScore between the BC subclasses. The infiltrating score of 10 immune cells was evaluated with the “MCPcounter” R package. The infiltrating scores of 28 immune cells were calculated with single-sample gene set enrichment analysis (ssGSEA) with the “gsva” R package [26].

### 2.4. Identification and Validation of the Prognostic Ferroptosis-Related Gene Signature

Ferroptosis-related genes that showed significance (*p* < 0.05) in both the Kaplan–Meier and Cox analyses were selected as potential prognostic genes. These genes were enrolled in a disease-free survival-based LASSO Cox regression model in the training cohort. The LASSO analysis was performed by applying the “glmnet” R package study to screen for the best penalty parameter lambda [27–29]. Risk score was calculated on the basis of the normalized gene expression level and regression coefficient of the corresponding gene as follows: risk score = sum (gene expression level × corresponding coefficient). Then, the patients were grouped into high- and low-risk groups according to their median risk scores. The concordance index (*C* index) for assessing the predictive accuracy of the six-gene model was obtained using the “risksetROC” R package. The survival difference between the two groups was measured using the Kaplan–Meier analysis. Cox and receiver-operating characteristic (ROC) analyses were also conducted for further assessment of the gene signature prognostic ability. Moreover, to verify the stability of the model obtained, the same formula and statistical methods were performed in TCGA test dataset and GEO cohort.

### 2.5. Expression Validation of the Prognostic Ferroptosis-Related Gene

Prognostic FRGs were validated with Kaplan–Meier survival curve in the GSE21653 cohort. Protein immunohistochemistry in normal human and tumor tissues was validated in the Human Protein Atlas (http://www.proteinatlas.org). To verify the expression profiles of prognostic FRGs in BC and normal tissues, we conducted the experimental validation using specimens from 10 BC patients who received esophagectomy between July 2020 and January 2021 in the Affiliated Hospital of Jiangnan University. Ten normal esophageal mucosal tissues were used as controls. The study was approved by the internal review board of the Affiliated Hospital of Jiangnan University. In terms of qRT-PCR, total RNA from normal breast samples (*n* = 10) and BC samples (*n* = 10) was isolated using the TRIzol reagent (Invitrogen, Carlsbad, CA, USA) in accordance with the manufacturer's instructions. Complementary DNA was synthesized from 1 *μ*g of total RNA using the PrimeScript RT reagent kit with a genomic DNA eraser (Takara). qRT-PCR was performed using SYBR Select Master Mix (Life Technologies, Austin, TX, USA) in a 7300 qRT-PCR system (Applied Biosystems, Foster City, CA, USA) using the following settings: 95°C for 2 min, followed by 40 cycles of 94°C for 20 s, 58°C for 20 s, and 72°C for 20 s. Glyceraldehyde-3-phosphate dehydrogenase (GAPDH) was used as the internal normalized reference to genes. The fold change was determined using the equation 2 − ΔΔCt (ΔΔCt = (ΔCt of genes of interest) − (ΔCt of GAPDH)). The primer sequences used are as follows: CARS1: F: 5′-CCATGCAGACTCCACCTTTAC-3′, R: 5′-GCAATACCACGTCACCTTTTTC-3′; CHAC1: F: 5′-GAACCCTGGTTACCTGGGC-3′, R: 5′-CGCAGCAAGTATTCAAGGTTGT-3′; FANCD2: F: 5′-AAAACGGGAGAGAGTCAGAATCA-3′, R: 5′-ACGCTCACAAGACAAAAGGCA-3′; AIFM2: F: 5′-AGACAGGGTTCGCCAAAAAGA-3′, R: 5′-CAGGTCTATCCCCACTACTAGC-3′; G6PD: F: 5′-CGAGGCCGTCACCAAGAAC-3′, R: 5′-GTAGTGGTCGATGCGGTAGA-3′; and HMOX1: F: 5′-AAGACTGCGTTCCTGCTCAAC-3′, R: 5′-AAAGCCCTACAGCAACTGTCG-3′.

### 2.6. Nomogram Development and Evaluation of Predictive Performance

To improve the predictive accuracy of the risk score model and provide a quantitative method for clinicians to predict the DFS of patients with BC, independent prognostic factors were identified on the basis of the patient's clinical information and risk score by performing a multivariate Cox regression analysis. Next, a nomogram was constructed using the survival rate and “RMS” R package, and a correction curve was drawn to evaluate the consistency between the actual and predicted recurrence rates. The predictive performance of the nomogram was assessed by discrimination and calibration. Moreover, the concordance (*C*) index ranged from 0.5 to 1.0. Values between 0.5 and 1.0 represent random opportunities and excellent ability to predict survival using this model.

### 2.7. Analysis of Biological Properties and Pathways Related to the Gene Signatures

KEGG pathway analyses were performed to annotate the biological characteristics of the ferroptosis-related gene signatures used to construct the risk models. TCGA expression profile was selected for ssGSEA using the “GSVA” R package to calculate the scores of each sample in different functions and to obtain the ssGSEA scores of each sample corresponding to each function. Furthermore, we calculated the correlation between these functions and risk scores and chose the KEGG pathway with a Pearson correlation coefficient > 0.4 and *p* value < 0.05.

### 2.8. Statistical Analysis

The Student *t*-test was applied to identify the differentially expressed FRGs between tumor and normal tissues and evaluate the differences in ImmuneScore, StromalScore, and ESTIMATEScore between the risk groups. The chi-square or Fisher exact test was used to compare the characteristics between the two groups. The difference in the ssGSEA score of immune cells or pathways between the risk groups was evaluated with the Mann–Whitney test, with *p* values adjusted with the Benjamini-Hochberg method. The OS or DFS between the groups was compared using the Kaplan–Meier analysis with the log-rank test. The independent predictors of DFS were identified using univariate and multivariate Cox regression analyses. All statistical analyses were performed with the R Version 3.6.3 or GraphPad Prism Version 8.0 software. All *p* values were two-tailed, and *p* values < 0.05 were considered statistically significant.

## 3. Results

**Figure 1:**
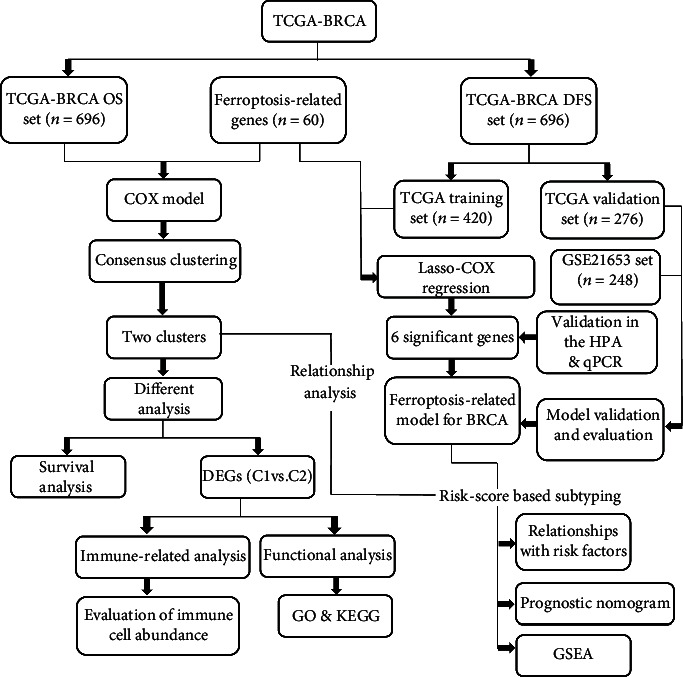
Flowchart of the data collection and analysis.

The detailed workflow of this study is shown in Figure 1. A total of 696 BC patients from the TCGA-BRCA cohort and 248 BC patients from the GSE21653 cohort were finally recruited. Of TCGA samples, 60% were randomly selected as the training set (420 BC samples), and the remaining 40% were allocated as the internal validation set (276 BC samples). The detailed corresponding clinical information of the three datasets is summarized in Table 1.

### 3.1. Classification of BC Based on FRGs

**Figure 2:**
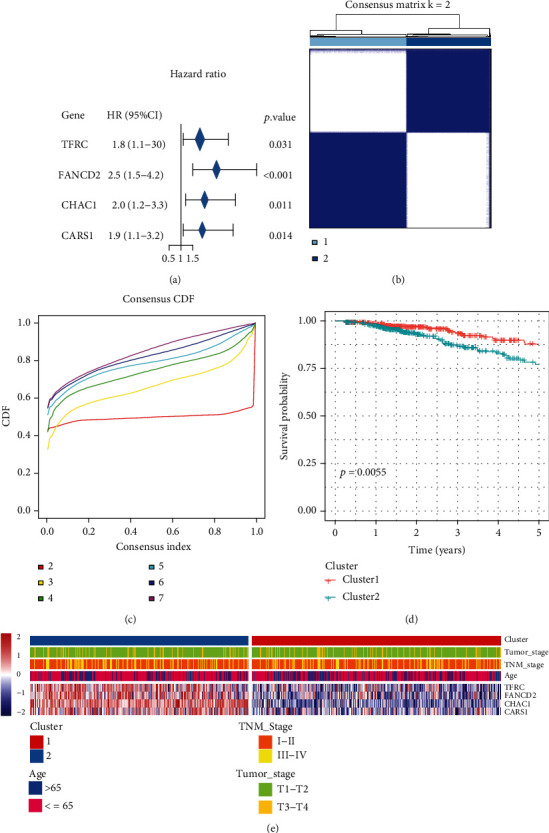
Identification of breast cancer subclasses using consensus clustering in the ferroptosis set. (a) Univariate Cox regression analysis. Forest plot of four significant ferroptosis-related genes associated with the overall survival in breast cancer in the TCGA-BRCA cohort. (b) Clustering using four ferroptosis-related genes. The patients were divided into clusters 1 and 2. (c) Empirical cumulative distribution function plot displaying consensus distributions for each *k*. (d) Survival analysis of the patients in clusters 1 and 2 in TCGA cohort. (e) The heat map shows the association of the clusters and clinical pathological features based on the four ferroptosis-related genes.

Four FRGs, namely, TFRC, FANCD2, CHAC1, and CARS1, were selected on the basis of the significant prognostic value with OS (*p* < 0.05) and subjected to consensus clustering analysis (Figure 2(a)). The “ConsensusClusterPlus” R package was used to divide the BC samples from the TCGA-BRCA into 2 different clusters. A comprehensive correlation coefficient was used to determine the optimal *k* value. Thereafter, the optimal total cluster number was set to *k* = 2 (with the two subclasses designated as clusters 1 and 2; Figures 2(b) and 2(c)). The OS rate was compared between the 2 clusters, and a significant difference was found (*p* = 0.005; Figure 2(d)) in TCGA cohort. The relationships between the four FRGs and clinical features were analyzed in TCGA cohort (Figure 2(e)).

### 3.2. Differential Expression between Clusters and Functional Enrichment Analysis

By using the “limma” R package with a FDR < 0.05 and logFC > 1.2, 485 mRNAs were identified as DEGs between clusters 1 and 2, including 305 upregulated and 180 downregulated genes. The volcano plot shows the fold change and statistical significance of the mRNA expression between the two clusters in Figure S1A. The heat map depicts the relative expression level of the top 100 upregulated and downregulated genes in Figure S1B.

The GO functional analysis of the potential target genes revealed 565 categories associated with biological processes, 68 cell component-associated categories, and 107 functional GO molecular function-associated categories forming the top 8 categories, respectively (Figures S2A–S2C). The KEGG functional analysis revealed 23 categories, and the top 8 categories are shown in Figure S2D.

**Figure 3:**
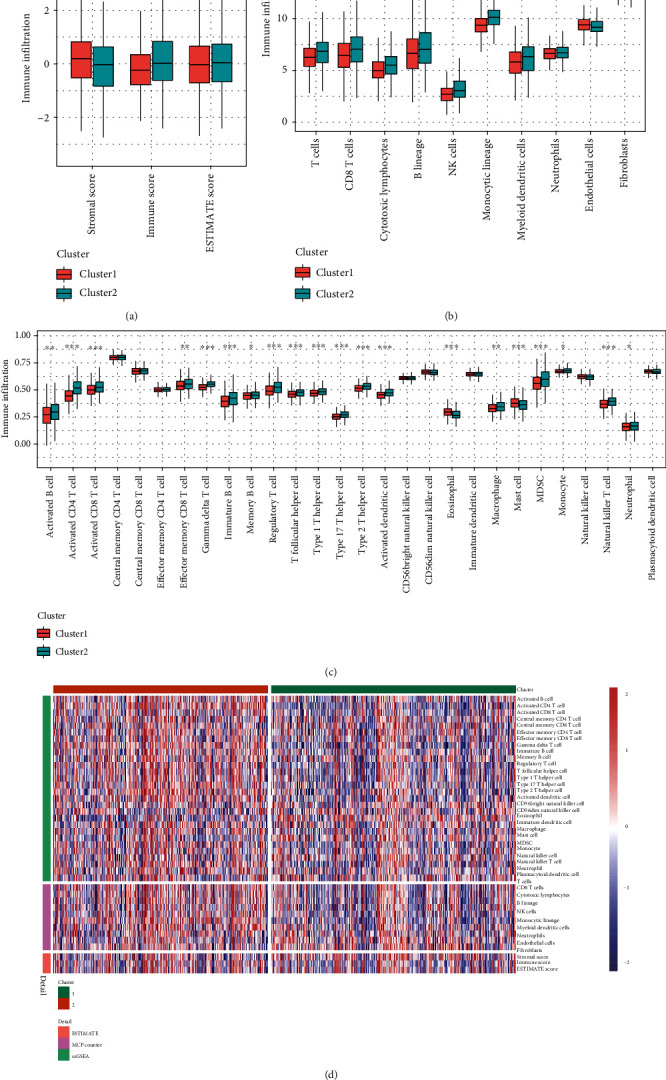
Comparison of StromalScore, ImmuneScore, and ESTIMATEScore between the two clusters of breast cancers in the TCGA-BRCA cohort (a). Evaluation of the infiltrating scores of 10 immune cells between the two clusters of breast cancer in the TCGA-BRCA cohort (b). Comparison of ssGSEA scores between the two clusters in TCGA cohort (c). The heat map shows the three immune scores between the two clusters (d). Adjusted *p* values: ^∗^*p* < 0.05; ^∗∗^*p* < 0.01; ^∗∗∗^*p* < 0.001.

StromalScore, ImmuneScore, and ESTIMATEScore were calculated using the “ESTIMATE” R package. The results showed that StromalScore was significantly higher in cluster 1 (*p* < 0.01), and ImmuneScore was higher in cluster 2 (*p* < 0.01), while no significant difference was observed in terms of ESTIMATEScore between the two clusters (Figure 3(a)). Ten immune cell scores were evaluated using the “MCPcounter” R package, and the results showed that the immune cell scores of T cells, CD8 T cells, cytotoxic lymphocytes, B lineage, NK cells, monocytic lineage, and myeloid dendritic cells were higher in cluster 2 than in cluster 1 (Figure 3(b)). The results of the ssGSEA demonstrated that only the eosinophil and mast cell immune scores were significantly higher in cluster 1 (*p* < 0.05). The immune scores of activated B cells, activated CD4 T cells, activated CD8 T cells, effector memory CD8 T cells, gamma delta T cells, immature B cells, memory B cells, regulatory T cells, T follicular helper cells, type 1 T helper cells, type 17 T helper cells, type 2 T helper cells, activated dendritic cells, macrophage, MDSC, monocyte, natural killer T cells, and neutrophils are higher in cluster 2 (*p* < 0.05; Figure 3(c)). A comparison of three immune score methods between molecular subtypes is shown in Figure 3(d) with a heat map.

### 3.3. Identification of the Prognostic Ferroptosis-Related Gene Signature

The Kaplan–Meier and univariate Cox analyses were conducted over the TCGA-BRCA training cohort for DFS, and 656 potential prognostic genes were identified. The potential prognostic genes and 60 FRGs were intersected to obtain a list containing six ferroptosis-related potential prognostic genes, including CARS1, CHAC1, FANCD2, AIFM2, G6PD, and HMOX1 (Figure S3). The six ferroptosis-related potential prognostic genes were then subjected to a DFS-based LASSO Cox regression model (Figure S4A). When six genes were gathered, the regression model reached its optimal ability (Figure S4B).

### 3.4. Relationships between the Expression of 6 FRGs and Important Clinical Characteristics

High expression of CARS1 has significant positive association with molecular subtype (*p* < 0.05) (Table S1). High expression of CHAC1 has close relationship with T stage, PR, ER, Her-2, menopause status, and molecular subtype (*p* < 0.05) (Table S2). High expression of FANCD2 has significant association with T, N stage, pathologic stage, PR, ER status, molecular subtype, and tumor location (*p* < 0.05) (Table S3). High expression of AIFM2 has significant association with T stage (*p* < 0.05) (Table S4). High expression of G6PD has close association with pathologic stage, Her-2 status, and molecular subtype (*p* < 0.05) (Table S5). High expression of HMOX1 has significant relationship with menopause status and molecular subtype (*p* < 0.05) (Table S6).

### 3.5. Construction Genes Weighted by Their Coefficients to Create a Ferroptosis-Related Prognosis Model in TCGA Cohort

By linearly combining the six FRGs weighted by their coefficients from the multivariate Cox analysis, a hazard model was constructed using the following formula:

(1) $$\begin{array}{c} \text{Risk score}=\left(E_{\text{CARS}1}\times 0.35\right)+\left(E_{\text{CHAC}1}\times 0.019\right)+\left(E_{\text{FANCD}2}\times 0.32\right)+\left(E_{\text{AIFM}2}\times -0.25\right)+\left(E_{\text{G}6\text{PD}}\times 0.076\right)+\left(E_{\text{HMOX}1}\times 0.18\right). \end{array}$$

*E*_CARS1_ is the expression value of the gene *CARS1*. The rest are similar to the gene *CARS1*.

**Figure 4:**
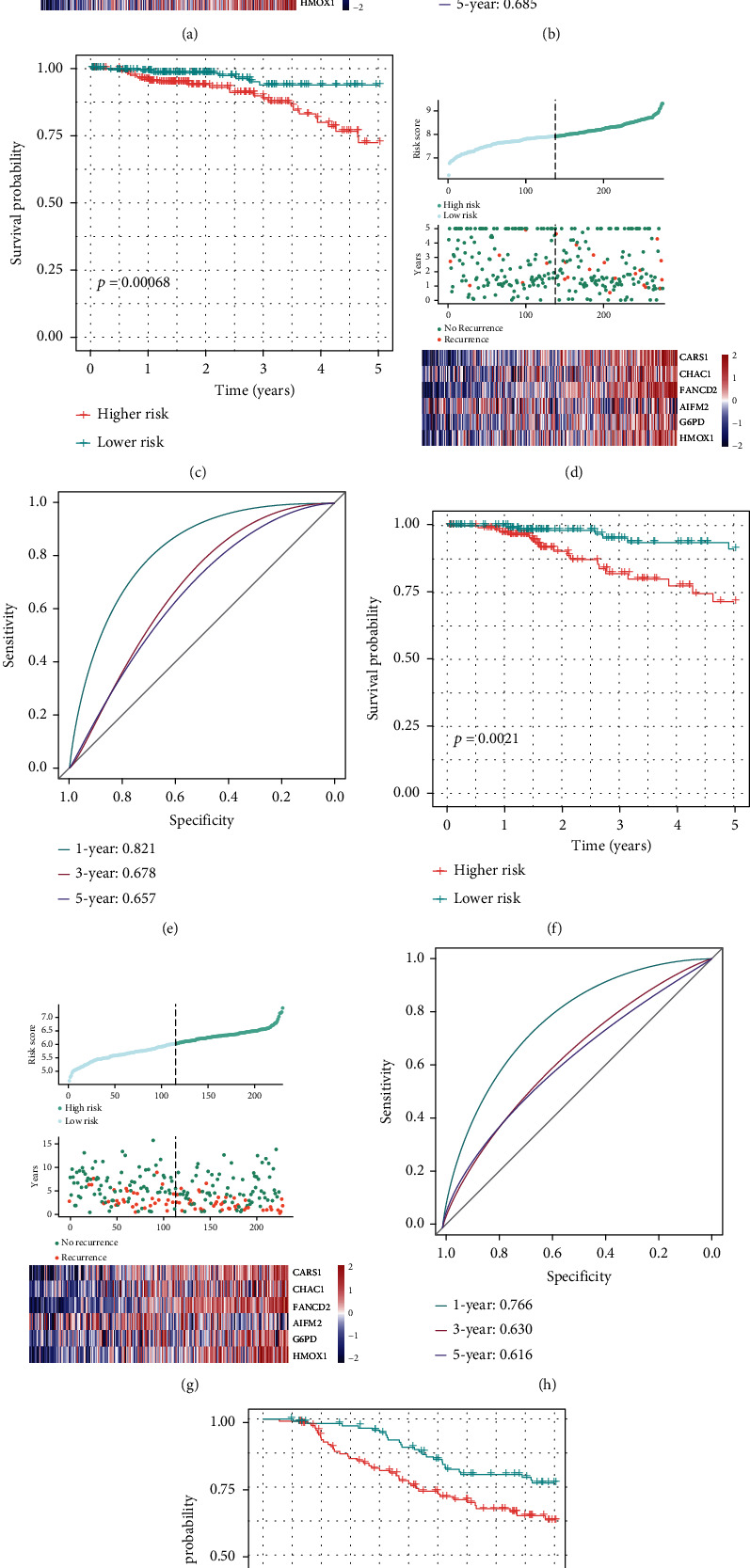
Construction of the prognostic prediction model and model validation. (a, d, g) Risk score (top), disease-free survival (middle) of the patients, and expression profiles of the six ferroptosis-related genes (bottom) in TCGA training, TCGA internal validation, and GSE21653 external validation sets. (b, e, h) The areas under the curve of the time-dependent ROC curves show and verified the prognostic performance of the risk scores in TCGA training, TCGA internal validation, and GSE21653 external validation sets. (c, f, i) Kaplan–Meier curves for disease-free survival in the high- and low-risk groups in TCGA training, TCGA internal validation, and GSE21653 external validation sets.

The risk score of each sample was calculated using the above-mentioned method. The patients in TCGA training cohort were divided into high- (*n* = 212) and low-risk groups (*n* = 208) according to the optimal cutoff value determined using the “survminer” R package. Detailed risk scores, survival information, and ferroptosis-related gene expressions are presented in Figure 4(a). The ROC analysis is shown in Figure 4(b), and the ROC curves reach 0.708, 0.626, and 0.685 at 1 year, 3 years, and 5 years, respectively. As shown in the Kaplan–Meier curves in Figure 4(c), the high-risk group had a higher probability of recurrence than the low-risk group (*p* < 0.001).

### 3.6. Validation of the Six-Ferroptosis-Gene Signatures Using the Test Dataset

The robustness of the model was examined in the test dataset from TCGA test cohort (*n* = 276), including 121 samples in the high-risk group and 155 samples in the low-risk group, using the same risk formula. The detailed risk scores, survival information, and ferroptosis-related gene expressions are also displayed (Figure 4(d)). The areas under the curve of the time-dependent ROC in 1 year, 3 years, and 5 years were 0.821, 0.678, and 0.657, respectively (Figure 4(e)). The patients in the high-risk group had a higher risk of recurrence than those in the low-risk group, consistent with the former results (Figure 4(f)).

To further test the robustness of the constructed model, the patients (*n* = 248) from the GEO21653 cohort were categorized into high- (113 samples) and low-risk groups (135 samples) according to the same risk formula as described earlier. Detailed risk scores, survival information, and ferroptosis-related gene expressions are presented in Figure 4(g). The ROC analysis is shown in Figure 4(h), and the ROC curves reached 0.766 at 1 year, 0.630 at 3 years, and 0.616 at 5 years. As demonstrated in the Kaplan–Meier curves in Figure 4(i), the high-risk group had a higher probability of recurrence than the low-risk group (*p* = 0.014).

### 3.7. Correlation of the Prognostic Risk Score with Pathological Features

**Figure 5:**
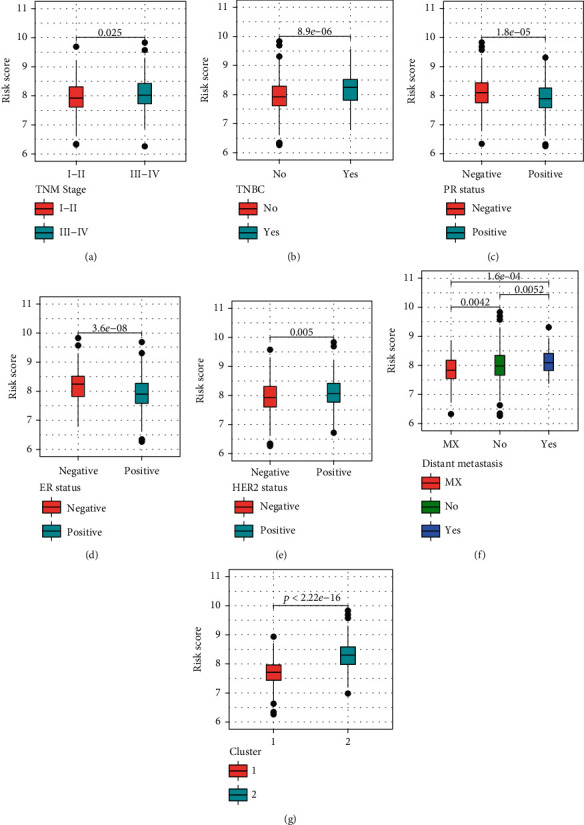
Relationships between the risk score model and important clinical characteristics. (a) TNM stage, (b) triple-negative status, (c) estrogen receptor (ER) status, (d) progesterone receptor (PR) status, (e) Her-2 status, (f) metastasis status at diagnosis, and (g) cluster group. The differences were compared using the Wilcoxon test.

Significant differences in risk score were found between the patients with TNM stage (*p* = 0.025), triple-negative status (*p* < 0.0001), estrogen receptor (ER) status (*p* < 0.0001), progesterone receptor (PR) status (*p* < 0.0001), Her-2 status (*p* = 0.005), metastasis status at diagnosis (*p* = 0.0052), and cluster group (*p* < 0.0001; Figures 5(a)–5(g)).

### 3.8. Survival Analysis Using Prognostic Risk Scores and Correlations with Pathological Features

The Kaplan–Meier analysis revealed that the DFS outcome in the high-risk group was worse than that in the low-risk group with positive lymph node metastasis (*p* = 0.0012), negative lymph node metastasis (*p* = 0.034), and distant metastasis at diagnosis (*p* < 0.001); T3–T4 tumor stage (*p* = 0.036); T1–T2 tumor stage (*p* < 0.0001); positive Her-2 status (*p* < 0.0001); positive ER status (*p* < 0.001); positive PR status (*p* < 0.001); triple-negative BC (TNBC; *p* = 0.0095); I-II TNM stage (*p* < 0.001); III–IV TNM stage (*p* = 0.02); cluster 1 (*p* = 0.0014); cluster 2 (*p* < 0.0001); age at diagnosis > 65 years (*p* = 0.0019); and age at diagnosis ≤ 65 years (*p* = 0.0031; Figures S5A– S5O).

### 3.9. Expression Validation of the Prognostic Ferroptosis-Related Gene

The Kaplan–Meier survival curve of 6 prognostic FRGs in the GSE21653 cohort for validation showed the same trend with training cohort (Figure S6).

**Figure 6:**
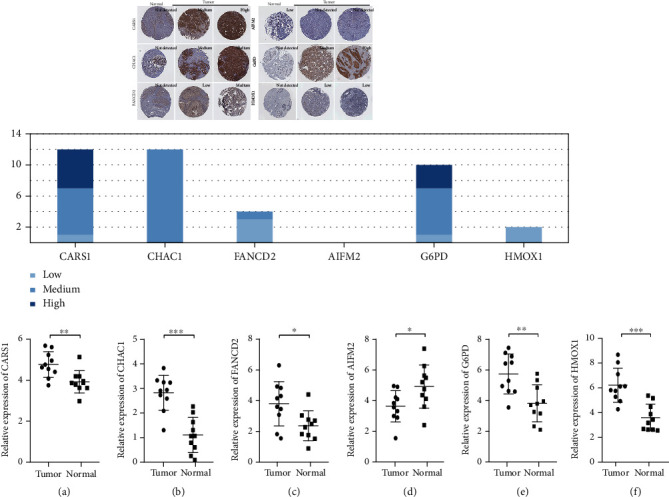
Expression levels of CARS1, CHAC1, FANCD2, AIFM2, G6PD, and HMOX1 in breast cancer and normal breast tissues validated in the Human Protein Atlas. The bar graph shows the cases with different expression levels of the six genes in breast cancer tissues. Light blue indicates low expression level; dark blue, low expression level; and shade between the two colors, medium expression level. The longitudinal axis shows the number of cases, and the transverse axis represents different genes. (a–e) Validation of the expression levels of the six genes between the normal breast samples (*n* = 10) and breast cancer samples (*n* = 10) by polymerase chain reaction analysis. All the data are presented as mean ± SD. Significant differences are defined by ^∗^*p* < 0.05, ^∗∗^*p* < 0.01, and ^∗∗∗^*p* < 0.001.

The AIFM2 protein expression was downregulated and the CARS1, CHAC1, FANCD2, G6PD, and HMOX1 protein expressions were upregulated in BC tissues as compared with normal tissues in the Human Protein Atlas (Figure 6).

To further verify the accuracy of the six-gene prognostic signature, we detected the expression levels of CARS1, CHAC1, FANCD2, AIFM2, G6PD, and HMOX1 in BRCA and adjacent tissues by using RT-PCR. Ten pairs of samples were used in the analysis. The experimental results revealed that the expression levels of CARS1, CHAC1, FANCD2, G6PD, and HMOX1 in the BRCA-positive patients were significantly upregulated, whereas those of AIFM2 were downregulated (Figures 6(a)–6(e)).

### 3.10. Univariate and Multivariate Cox Analyses of Prognostic Risk Scores and Individualized Prognostic Prediction Models

**Figure 7:**
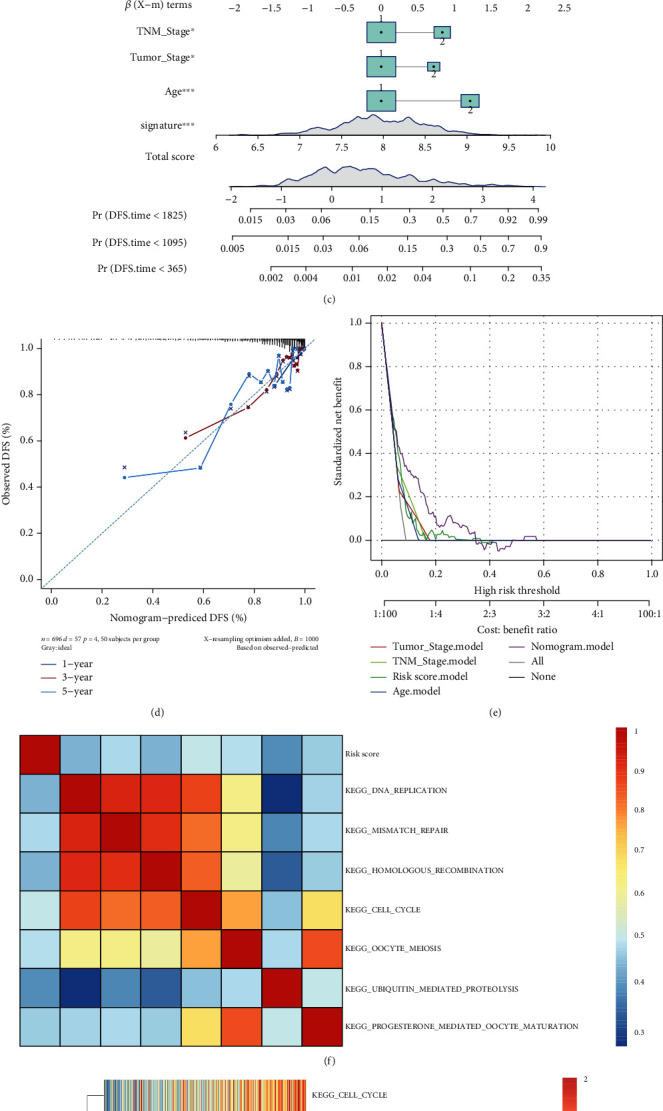
Results of the univariate and multivariate Cox regression analyses regarding disease-free survival in TCGA cohort (a, b). A nomogram of the breast cancer cohort (training set) was used to predict the disease-free survival (c). Calibration maps were used to predict 1-, 3-, and 5-year survival times (d). A decision curve analysis was used to compare the clinical usefulness of nomography with that of age, TNM stage, and tumor stage based on the threshold probability (e). A gene set enrichment analysis was performed. (f) The heat map shows the correlation between the risk scores and the top 7 KEGG pathways and the relationships among the KEGG pathways (correlation coefficient > 0.4 and *p* < 0.05). (g) Clustering of 7 KEGG pathways and the heat map show the risk score of each case in the top 7 KEGG pathways. The horizontal axis represents the breast cancer samples, and the risk score increases from left to right.

Univariate and multivariate Cox regression analyses were performed on datasets combined with TCGA-BRCA and GSE21653. The univariate Cox regression analysis revealed that the risk scores, age at diagnosis, PR status, ER status, Her-2 status, tumor stage, TNBC, lymph node metastasis, and cluster were associated with the DFS rate of the BC patients (*p* < 0.05; Figure 7(a)). The multivariate Cox regression analysis revealed that the risk scores, age at diagnosis, TNM stage, and tumor stage were the independent risk factors for predicting the DFS rate of the BC patients (*p* < 0.001; Figure 7(b)).

By using the synthesis of the six-ferroptosis-related-gene signature, a nomogram was generated on the basis of age, TNM stage, tumor stage, and risk score to predict the probability of 1-, 3-, and 5-year DFS rates. Several factors were scored on the basis of the proportion of the contribution to the recurrence risk as shown in Figure 7(c). The calibration curve results show that the predicted survival rate is closely related to the actual recurrence rate (Figure 7(d)). Furthermore, a decision curve analysis was used to compare the clinical usefulness of nomography with that of age, TNM stage, and tumor stage based on the threshold probability.  Figure 7(e) shows that the nomogram is an excellent predictive evaluation model and superior to risk score, age, TNM stage, or tumor stage level alone.

### 3.11. Gene Set Enrichment Analysis

Seven KEGG pathway signals with Pearson correlation coefficients > 0.4 and *p* values < 0.05 were selected, including DNA replication, mismatch repair, homologous recombination, cell cycle, oocyte meiosis, ubiquitin-mediated proteolysis, and progesterone-mediated oocyte maturation. The Pearson correlation coefficient between the risk scores and the KEGG pathway signals is shown in Figure 7(f).  Figure 7(g) shows the change in the ssGSEA score of the KEGG pathway in each sample with increased risk score.

## 4. Discussion

In our study, 60 FRGs were extracted from previous studies [22–25]. We discovered that these FRGs could dichotomize BC patients into high- and low-risk groups for discrimination of OS. The functional analyses of DEGs between the two subgroups also revealed significant differences in immune-related pathways, including the chemokine and IL-17 signaling pathways, which warrants further investigation of the potential association between immunity and ferroptosis in BC. Therefore, ESTIMATE was used to quantify immune cell infiltration and the stromal component between the low- and high-risk groups. We observed that the high-risk group had higher ImmuneScores, lower StromalScores, and similar ESTIMATEScores, which suggest that the high-risk group had higher levels of immune cell infiltration in the tumor microenvironment. Further exploration revealed that the numbers of activated dendritic cells (aDCs), macrophage, type 1 T helper cells (Th1), activated CD8 T+ cells, regulatory T (Treg) cells, and neutrophils were remarkably higher in the high-risk group on the basis of the enrichment scores of ssGSEA in the TCGA-BRCA cohort, whereas the numbers of eosinophils and mast cells were significantly higher in the low-risk group. These differences may imply the sophisticated relationships between ferroptosis and immunity.

Previous studies demonstrated that higher amounts of Treg cells, macrophages, and neutrophils usually tend to be associated with poorer prognosis in some solid tumors [30–33], which is consistent with our study. We observed that only eosinophils and mast cells had higher proportions in the low-risk BC patients. Mast cells, important natural immune guard with high functional plasticity, are associated with prolonged patient survival and inhibition of cancer progression [34]. Mast cells could regulate inhibitory immune response to stimulate tumor immune activity and maintain the balance of the tumor microenvironment similar to the function of programmed cell death 1 (PD-1) and programmed cell death ligand 1 (PD-L1) [35]. Thus, mast cells may be another potential treatment target to enhance the immune response to various stimuli, including signals and components from the tumor microbiota. Eosinophils, an antitumor immune system independent of T cells, could kill cancer cells directly or suppress the growth of tumor by secreting TNF-*α* and IL-18 [36]. A study of Hollande et al. also found that the increased IL-33 expression level in hepatocellular carcinoma tissue contributes to the differentiation and proliferation of eosinophils and promotes the expression of chemokine CCL11 in cancer cells, inducing the recruitment of eosinophils into tumors and subsequent inhibition of tumor growth [37]. In several clinical studies, increased peripheral-blood eosinophils were associated with better prognosis in patients who received CTLA-4 immunotherapy [36, 38]. We may consider that the combination of T cell- and eosinophil-targeted immunotherapy may open a new avenue for anticancer treatment strategy in solid tumors. On the basis of this study, the underlying interaction mechanism between ferroptosis and mast cells or eosinophils should be investigated further.

Long survival times have been achieved in BC, as the improvement of the comprehensive treatment of local recurrence or distant metastasis has become the main challenge for clinicians. Previous study has built a ferroptosis score model and showed a good predictive value for OS in BS patients [39]. In this study, a novel prognostic model for DFS combined with 6 FRGs was also established with the TCGA-BRCA training cohort and revealed a good predictive value of recurrence in internal and external validation cohorts. These 6 genes could be roughly divided into three categories, including (anti) oxidant metabolism (CARS1, CHAC1, and HMOX1), energy metabolism (AIFM2 and G6PD), and DNA damage repair (FANCD2). Limited studies about CARS1 (cysteinyl-tRNA synthetase) have been reported so far. A previous study revealed that the knockdown of CARS1 could activate serine biosynthesis and transsulfuration and inhibit ferroptosis by prohibiting the induction of lipid-based ROS [40]. CARS1 was also recruited into a multigene signature to predict the prognosis in esophageal adenocarcinoma and hepatocellular carcinoma [11, 14]. CHAC1 (ChaC glutathione-specific gamma-glutamyl cyclotransferase 1) degradation of glutathione contributes to ferroptosis induced by cystine starvation in TNBC cells via the GCN2-eIF2*α*-ATF4 pathway [41]. Previous studies demonstrated that HMOX1 (heme oxygenase-1), a well-known antioxidant enzyme, could promote the ferroptosis of tumor cells in breast and renal cancers by involving in iron supplement and lipid peroxidation [42, 43]. HMOX1 plays important anticancer, anti-inflammatory, antiapoptotic, antiproliferative, and antioxidant roles [44]. The underlying interaction mechanism between HMOX1 and ferroptosis warrants investigation. G6PD (glucose-6-phosphate dehydrogenase) and AIFM2 (apoptosis-inducing factor mitochondrial-associated 2) are ferroptosis regulators related to energy metabolism. G6PD was reported to inhibit erastin-induced ferroptosis when knocked down in non-small-cell lung cancer cells by reducing ROS directly under the pentose phosphate pathway [45]. Other studies also found that high G6PD expression level was significantly associated with poor prognosis in bladder and colorectal cancers [46, 47]. AIFM2, also known as FSP1 (ferroptosis suppressor protein 1), is considered the key regulator of apoptosis, and overexpression of AIFM2 induces apoptosis and reduces cell sensitivity to ferroptosis [24, 25]. Several studies have shown that AIFM2 translocation could promote the apoptosis of breast, gastric, and liver cancer cells in a caspase-independent manner [48, 49]. ASAP1 (ArfGAP with SH3 domain, ankyrin repeat, and PH domain 1) overexpression could promote the progression of triple-negative BC by regulating AIFM2 in apoptosis-related signaling pathway [50]. FANCD2 (fanconi anemia complementation group D2), like BRCA2, may play an important role in the recombination DNA repair pathways [51]. Wang et al. reported that inhibition of FANCD2 could induce DNA damage and suppress lung cancer progression [52]. A risk model for predicting therapeutic responses obtained better predictive efficiency when combined with FANCD2 expression and tumor mutation burden in lung cancer [53]. In summary, five of the genes (CARS1, CHAC1, FANCD2, G6PD, and HMOX1) in the prognostic model have been reported to contribute to ferroptosis and to be upregulated in BC tumor tissue, in contrast to AIFM2. Whether these genes play a role in the prognosis of BC patients by influencing ferroptosis remains to be elucidated owing to the limited associated reports on these genes. The KEGG enrichment analysis in our study showed two main potential pathways, including DNA replication and mismatch repair. Further basic experiment validations are needed.

Several limitations exist in this study. First, cluster identification and prognostic model establishment and validation were conducted with retrospective data from a public database. Therefore, real-world data should be collected and used to verify the clinical usefulness of our prediction model. The limited meaningful clinical characteristics provided in public databases might have reduced the efficiency of our prediction model, although we tried to minimize the risk by performing multivariate Cox regression analyses. Second, only 60 FRGs were recruited in this study. The possibilities that other genes in the signature may be more strongly related to other pathways in BC and that more ferroptosis regulators have been identified owing to the rapid emergence of new studies on ferroptosis are undeniable. Third, the correlations between risk and biological function in BC warrant experimental investigation. The six ferroptosis-related gene markers identified in this study may be potential prognostic biomarkers that provide new insight into the research and treatment of BC.

In summary, in this study, we found that the FRGs could be used to classify BC patients according to different clinical and molecular features. A novel prognostic model with the six FRGs was established and showed a good predictive value of recurrence in the derivation and validation BC cohorts. However, the applicability of this model still needs validation in clinical research with large size examples. What is more, the potential mechanisms between FRGs and biological function in BC remain rarely known and need further exploration.

## Figures and Tables

**Table 1:** Clinical characteristics of the BC patients used in this study.

Characteristic	TCGA [*n* = 696] (total)	TCGA [*n* = 420] (training set)	TCGA [*n* = 276] (internal validation set)	GSE21653 [*n* = 248] (external validation set)
Age (years)				
≤65	498	306	192	186
>65	198	114	84	62
Tumor stage				
T1-T2	589	358	231	178
T3-T4	107	62	45	63
Lymph node metastasis				
Yes	355	209	146	116
No	366	209	127	130
Unknown	5	2	3	
TNM stage				
I-II	524	326	198	NA
III-IV	172	94	78	NA
Distant metastasis				
Yes	9	6	3	NA
No	598	362	236	NA
Unknown	89	52	37	NA
Estrogen receptor status				
Negative	159	325	212	107
Positive	537	95	64	139
Progesterone receptor status				
Negative	232	278	186	122
Positive	464	142	90	124
Her-2 overexpression				
Negative	539	319	220	NA
Positive	157	101	56	NA
Triple negative				
Yes	112	70	42	72
No	584	350	234	176
OS status				
Survival	619	374	245	NA
Censored	77	46	31	NA
DFS status				
Disease-free	603	361	242	169
Disease	93	59	34	79

## Data Availability

The data that support the findings of this study are available from the corresponding author upon reasonable request.

## Supplementary Materials

Supplementary Materials Figure S1: identification of differentially expressed mRNAs between clusters 1 and 2 in the TCGA-BRCA cohort. (A) Volcano plot. (B) Heat map. Figure S2: the Gene Ontology annotation of differentially expressed genes. GO enrichment: (A) BP, (B) CC, and (C) MF. (D) KEGG enrichment. Figure S3: the Kaplan–Meier curves show the six FRGs in the TCGA-BRCA training cohort. (A) CARS1, (B) CHAC1, (C) FANCD2, (D) AIFM2, (E) G6PD, and (F) HMOX1. Figure S4: construction of a six-gene signature model in the TCGA-BRCA training cohort. (A) LASSO coefficient profiles of the expressions of the candidate genes. (B) Selection of the penalty parameter (*λ*) in the LASSO model via sixfold cross-validation. Figure S5: stratified analysis in the whole TCGA-BRCA set. (A, B) Lymph node metastasis. (C) Distant metastasis at diagnosis. (D, E) Tumor stage. (F) Positive Her-2 status. (G) Positive ER status. (H) Positive PR status. (I) Triple-negative breast cancer. (J, K) TNM stage. (L, M) Cluster state. (N, O) Age at diagnosis. Figure S6: the Kaplan–Meier curves show the six FRGs in the GSE21653 cohort. (A) CARS1, (B) CHAC1, (C) FANCD2, (D) AIFM2, (E) G6PD, and (F) HMOX1. Table S1: relationships between the expression of CARS1 and important clinical characteristics. Table S2: relationships between the expression of CHAC1 and important clinical characteristics. Table S3: relationships between the expression of FANCD2 and important clinical characteristics. Table S4: relationships between the expression of AIFM2 and important clinical characteristics. Table S5: relationships between the expression of G6PD and important clinical characteristics. Table S6: relationships between the expression of HMOX1 and important clinical characteristics.
