# Supplementary material for: Correlation between Ferroptosis-Related Gene Signature and Immune Landscape, Prognosis in Breast Cancer
Source: J Immunol Res. 2022 Oct 11;2022:6871518. doi: 10.1155/2022/6871518 (PMC9613394; doi:10.1155/2022/6871518)
Supplement: Supplementary Materials — Figure S1: identification of differentially expressed mRNAs between clusters 1 and 2 in the TCGA-BRCA cohort. (A) Volcano plot. (B) Heat map. Figure S2: the Gene Ontology annotation of differentially expressed genes. GO enrichment: (A) BP, (B) CC, and (C) MF. (D) KEGG enrichment. Figure S3: the Kaplan–Meier curves show the six FRGs in the TCGA-BRCA training cohort. (A) CARS1, (B) CHAC1, (C) FANCD2, (D) AIFM2, (E) G6PD, and (F) HMOX1. Figure S4: construction of a six-gene signature model in the TCGA-BRCA training cohort. (A) LASSO coefficient profiles of the expressions of the candidate genes. (B) Selection of the penalty parameter (λ) in the LASSO model via sixfold cross-validation. Figure S5: stratified analysis in the whole TCGA-BRCA set. (A, B) Lymph node metastasis. (C) Distant metastasis at diagnosis. (D, E) Tumor stage. (F) Positive Her-2 status. (G) Positive ER status. (H) Positive PR status. (I) Triple-negative breast cancer. (J, K) TNM stage. (L, M) Cluster state. (N, O) Age at diagnosis. Figure S6: the Kaplan–Meier curves show the six FRGs in the GSE21653 cohort. (A) CARS1, (B) CHAC1, (C) FANCD2, (D) AIFM2, (E) G6PD, and (F) HMOX1. Table S1: relationships between the expression of CARS1 and important clinical characteristics. Table S2: relationships between the expression of CHAC1 and important clinical characteristics. Table S3: relationships between the expression of FANCD2 and important clinical characteristics. Table S4: relationships between the expression of AIFM2 and important clinical characteristics. Table S5: relationships between the expression of G6PD and important clinical characteristics. Table S6: relationships between the expression of HMOX1 and important clinical characteristics. [file 6871518.f1.zip › Table S1.docx]

Table S1. Relationships between the expression of CARS1 and important clinical characteristics.

| Characteristic | Low expression of CARS1 | High expression of CARS1 | p |
| --- | --- | --- | --- |
| T stage, n (%) |  |  | 0.315 |
| T1 | 152 (14.1%) | 125 (11.6%) |  |
| T2 | 304 (28.1%) | 325 (30.1%) |  |
| T3 | 67 (6.2%) | 72 (6.7%) |  |
| T4 | 17 (1.6%) | 18 (1.7%) |  |
| N stage, n (%) |  |  | 0.565 |
| N0 | 261 (24.5%) | 253 (23.8%) |  |
| N1 | 181 (17%) | 177 (16.6%) |  |
| N2 | 51 (4.8%) | 65 (6.1%) |  |
| N3 | 36 (3.4%) | 40 (3.8%) |  |
| M stage, n (%) |  |  | 0.783 |
| M0 | 457 (49.6%) | 445 (48.3%) |  |
| M1 | 9 (1%) | 11 (1.2%) |  |
| Pathologic stage, n (%) |  |  | 0.379 |
| Stage I | 96 (9.1%) | 85 (8%) |  |
| Stage II | 315 (29.7%) | 304 (28.7%) |  |
| Stage III | 110 (10.4%) | 132 (12.5%) |  |
| Stage IV | 8 (0.8%) | 10 (0.9%) |  |
| PR status, n (%) |  |  | 0.456 |
| Negative | 166 (16.1%) | 176 (17%) |  |
| Indeterminate | 3 (0.3%) | 1 (0.1%) |  |
| Positive | 351 (33.9%) | 337 (32.6%) |  |
| ER status, n (%) |  |  | 0.709 |
| Negative | 115 (11.1%) | 125 (12.1%) |  |
| Indeterminate | 1 (0.1%) | 1 (0.1%) |  |
| Positive | 404 (39%) | 389 (37.6%) |  |
| HER2 status, n (%) |  |  | 0.184 |
| Negative | 277 (38.1%) | 281 (38.7%) |  |
| Indeterminate | 3 (0.4%) | 9 (1.2%) |  |
| Positive | 72 (9.9%) | 85 (11.7%) |  |
| Molecular subtype, n (%) |  |  | < 0.001 |
| Others | 26 (2.4%) | 14 (1.3%) |  |
| LumA | 310 (28.6%) | 252 (23.3%) |  |
| LumB | 81 (7.5%) | 123 (11.4%) |  |
| Her2 | 38 (3.5%) | 44 (4.1%) |  |
| Triple negative | 86 (7.9%) | 109 (10.1%) |  |
| Menopause status, n (%) |  |  | 0.315 |
| Pre | 105 (10.8%) | 124 (12.8%) |  |
| Peri | 20 (2.1%) | 20 (2.1%) |  |
| Post | 363 (37.3%) | 340 (35%) |  |
| Tumor location, n (%) |  |  | 0.878 |
| Left | 283 (26.1%) | 280 (25.9%) |  |
| Right | 258 (23.8%) | 262 (24.2%) |  |
