# Supplementary material for: Correlation between Ferroptosis-Related Gene Signature and Immune Landscape, Prognosis in Breast Cancer
Source: J Immunol Res. 2022 Oct 11;2022:6871518. doi: 10.1155/2022/6871518 (PMC9613394; doi:10.1155/2022/6871518)
Supplement: Supplementary Materials — Figure S1: identification of differentially expressed mRNAs between clusters 1 and 2 in the TCGA-BRCA cohort. (A) Volcano plot. (B) Heat map. Figure S2: the Gene Ontology annotation of differentially expressed genes. GO enrichment: (A) BP, (B) CC, and (C) MF. (D) KEGG enrichment. Figure S3: the Kaplan–Meier curves show the six FRGs in the TCGA-BRCA training cohort. (A) CARS1, (B) CHAC1, (C) FANCD2, (D) AIFM2, (E) G6PD, and (F) HMOX1. Figure S4: construction of a six-gene signature model in the TCGA-BRCA training cohort. (A) LASSO coefficient profiles of the expressions of the candidate genes. (B) Selection of the penalty parameter (λ) in the LASSO model via sixfold cross-validation. Figure S5: stratified analysis in the whole TCGA-BRCA set. (A, B) Lymph node metastasis. (C) Distant metastasis at diagnosis. (D, E) Tumor stage. (F) Positive Her-2 status. (G) Positive ER status. (H) Positive PR status. (I) Triple-negative breast cancer. (J, K) TNM stage. (L, M) Cluster state. (N, O) Age at diagnosis. Figure S6: the Kaplan–Meier curves show the six FRGs in the GSE21653 cohort. (A) CARS1, (B) CHAC1, (C) FANCD2, (D) AIFM2, (E) G6PD, and (F) HMOX1. Table S1: relationships between the expression of CARS1 and important clinical characteristics. Table S2: relationships between the expression of CHAC1 and important clinical characteristics. Table S3: relationships between the expression of FANCD2 and important clinical characteristics. Table S4: relationships between the expression of AIFM2 and important clinical characteristics. Table S5: relationships between the expression of G6PD and important clinical characteristics. Table S6: relationships between the expression of HMOX1 and important clinical characteristics. [file 6871518.f1.zip › Table S2.docx]

Table S2. Relationships between the expression of CHAC1 and important clinical characteristics.

| Characteristic | Low expression of CHAC1 | High expression of CHAC1 | p |
| --- | --- | --- | --- |
| T stage, n (%) |  |  | 0.002 |
| T1 | 166 (15.4%) | 111 (10.3%) |  |
| T2 | 289 (26.8%) | 340 (31.5%) |  |
| T3 | 69 (6.4%) | 70 (6.5%) |  |
| T4 | 16 (1.5%) | 19 (1.8%) |  |
| N stage, n (%) |  |  | 0.054 |
| N0 | 260 (24.4%) | 254 (23.9%) |  |
| N1 | 191 (18%) | 167 (15.7%) |  |
| N2 | 51 (4.8%) | 65 (6.1%) |  |
| N3 | 29 (2.7%) | 47 (4.4%) |  |
| M stage, n (%) |  |  | 0.293 |
| M0 | 446 (48.4%) | 456 (49.5%) |  |
| M1 | 7 (0.8%) | 13 (1.4%) |  |
| Pathologic stage, n (%) |  |  | 0.075 |
| Stage I | 105 (9.9%) | 76 (7.2%) |  |
| Stage II | 307 (29%) | 312 (29.4%) |  |
| Stage III | 112 (10.6%) | 130 (12.3%) |  |
| Stage IV | 7 (0.7%) | 11 (1%) |  |
| PR status, n (%) |  |  | < 0.001 |
| Negative | 91 (8.8%) | 251 (24.3%) |  |
| Indeterminate | 3 (0.3%) | 1 (0.1%) |  |
| Positive | 418 (40.4%) | 270 (26.1%) |  |
| ER status, n (%) |  |  | < 0.001 |
| Negative | 39 (3.8%) | 201 (19.4%) |  |
| Indeterminate | 1 (0.1%) | 1 (0.1%) |  |
| Positive | 472 (45.6%) | 321 (31%) |  |
| HER2 status, n (%) |  |  | 0.036 |
| Negative | 278 (38.2%) | 280 (38.5%) |  |
| Indeterminate | 6 (0.8%) | 6 (0.8%) |  |
| Positive | 60 (8.3%) | 97 (13.3%) |  |
| Molecular subtype, n (%) |  |  | < 0.001 |
| Others | 16 (1.5%) | 24 (2.2%) |  |
| LumA | 380 (35.1%) | 182 (16.8%) |  |
| LumB | 104 (9.6%) | 100 (9.2%) |  |
| Her2 | 15 (1.4%) | 67 (6.2%) |  |
| Triple negative | 26 (2.4%) | 169 (15.6%) |  |
| Menopause status, n (%) |  |  | 0.018 |
| Pre | 102 (10.5%) | 127 (13.1%) |  |
| Peri | 14 (1.4%) | 26 (2.7%) |  |
| Post | 369 (38%) | 334 (34.4%) |  |
| Tumor location, n (%) |  |  | 0.412 |
| Left | 274 (25.3%) | 289 (26.7%) |  |
| Right | 267 (24.7%) | 253 (23.4%) |  |
