# Supplementary material for: Correlation between Ferroptosis-Related Gene Signature and Immune Landscape, Prognosis in Breast Cancer
Source: J Immunol Res. 2022 Oct 11;2022:6871518. doi: 10.1155/2022/6871518 (PMC9613394; doi:10.1155/2022/6871518)
Supplement: Supplementary Materials — Figure S1: identification of differentially expressed mRNAs between clusters 1 and 2 in the TCGA-BRCA cohort. (A) Volcano plot. (B) Heat map. Figure S2: the Gene Ontology annotation of differentially expressed genes. GO enrichment: (A) BP, (B) CC, and (C) MF. (D) KEGG enrichment. Figure S3: the Kaplan–Meier curves show the six FRGs in the TCGA-BRCA training cohort. (A) CARS1, (B) CHAC1, (C) FANCD2, (D) AIFM2, (E) G6PD, and (F) HMOX1. Figure S4: construction of a six-gene signature model in the TCGA-BRCA training cohort. (A) LASSO coefficient profiles of the expressions of the candidate genes. (B) Selection of the penalty parameter (λ) in the LASSO model via sixfold cross-validation. Figure S5: stratified analysis in the whole TCGA-BRCA set. (A, B) Lymph node metastasis. (C) Distant metastasis at diagnosis. (D, E) Tumor stage. (F) Positive Her-2 status. (G) Positive ER status. (H) Positive PR status. (I) Triple-negative breast cancer. (J, K) TNM stage. (L, M) Cluster state. (N, O) Age at diagnosis. Figure S6: the Kaplan–Meier curves show the six FRGs in the GSE21653 cohort. (A) CARS1, (B) CHAC1, (C) FANCD2, (D) AIFM2, (E) G6PD, and (F) HMOX1. Table S1: relationships between the expression of CARS1 and important clinical characteristics. Table S2: relationships between the expression of CHAC1 and important clinical characteristics. Table S3: relationships between the expression of FANCD2 and important clinical characteristics. Table S4: relationships between the expression of AIFM2 and important clinical characteristics. Table S5: relationships between the expression of G6PD and important clinical characteristics. Table S6: relationships between the expression of HMOX1 and important clinical characteristics. [file 6871518.f1.zip › Table S3.docx]

Table S3. Relationships between the expression of FANCD2 and important clinical characteristics.

| Characteristic | Low expression of FANCD2 | High expression of FANCD2 | p |
| --- | --- | --- | --- |
| T stage, n (%) |  |  | < 0.001 |
| T1 | 167 (15.5%) | 110 (10.2%) |  |
| T2 | 279 (25.8%) | 350 (32.4%) |  |
| T3 | 80 (7.4%) | 59 (5.5%) |  |
| T4 | 15 (1.4%) | 20 (1.9%) |  |
| N stage, n (%) |  |  | 0.046 |
| N0 | 266 (25%) | 248 (23.3%) |  |
| N1 | 172 (16.2%) | 186 (17.5%) |  |
| N2 | 48 (4.5%) | 68 (6.4%) |  |
| N3 | 46 (4.3%) | 30 (2.8%) |  |
| M stage, n (%) |  |  | 0.992 |
| M0 | 430 (46.6%) | 472 (51.2%) |  |
| M1 | 9 (1%) | 11 (1.2%) |  |
| Pathologic stage, n (%) |  |  | 0.001 |
| Stage I | 114 (10.8%) | 67 (6.3%) |  |
| Stage II | 286 (27%) | 333 (31.4%) |  |
| Stage III | 123 (11.6%) | 119 (11.2%) |  |
| Stage IV | 9 (0.8%) | 9 (0.8%) |  |
| PR status, n (%) |  |  | < 0.001 |
| Negative | 126 (12.2%) | 216 (20.9%) |  |
| Indeterminate | 2 (0.2%) | 2 (0.2%) |  |
| Positive | 391 (37.8%) | 297 (28.7%) |  |
| ER status, n (%) |  |  | < 0.001 |
| Negative | 76 (7.3%) | 164 (15.8%) |  |
| Indeterminate | 0 (0%) | 2 (0.2%) |  |
| Positive | 443 (42.8%) | 350 (33.8%) |  |
| HER2 status, n (%) |  |  | 0.124 |
| Negative | 288 (39.6%) | 270 (37.1%) |  |
| Indeterminate | 4 (0.6%) | 8 (1.1%) |  |
| Positive | 69 (9.5%) | 88 (12.1%) |  |
| Molecular subtype, n (%) |  |  | < 0.001 |
| Others | 33 (3%) | 7 (0.6%) |  |
| LumA | 390 (36%) | 172 (15.9%) |  |
| LumB | 45 (4.2%) | 159 (14.7%) |  |
| Her2 | 25 (2.3%) | 57 (5.3%) |  |
| Triple negative | 48 (4.4%) | 147 (13.6%) |  |
| Menopause status, n (%) |  |  | 0.818 |
| Pre | 113 (11.6%) | 116 (11.9%) |  |
| Peri | 19 (2%) | 21 (2.2%) |  |
| Post | 360 (37%) | 343 (35.3%) |  |
| Tumor location, n (%) |  |  | 0.012 |
| Left | 260 (24%) | 303 (28%) |  |
| Right | 281 (25.9%) | 239 (22.1%) |  |
