# Supplementary material for: Correlation between Ferroptosis-Related Gene Signature and Immune Landscape, Prognosis in Breast Cancer
Source: J Immunol Res. 2022 Oct 11;2022:6871518. doi: 10.1155/2022/6871518 (PMC9613394; doi:10.1155/2022/6871518)
Supplement: Supplementary Materials — Figure S1: identification of differentially expressed mRNAs between clusters 1 and 2 in the TCGA-BRCA cohort. (A) Volcano plot. (B) Heat map. Figure S2: the Gene Ontology annotation of differentially expressed genes. GO enrichment: (A) BP, (B) CC, and (C) MF. (D) KEGG enrichment. Figure S3: the Kaplan–Meier curves show the six FRGs in the TCGA-BRCA training cohort. (A) CARS1, (B) CHAC1, (C) FANCD2, (D) AIFM2, (E) G6PD, and (F) HMOX1. Figure S4: construction of a six-gene signature model in the TCGA-BRCA training cohort. (A) LASSO coefficient profiles of the expressions of the candidate genes. (B) Selection of the penalty parameter (λ) in the LASSO model via sixfold cross-validation. Figure S5: stratified analysis in the whole TCGA-BRCA set. (A, B) Lymph node metastasis. (C) Distant metastasis at diagnosis. (D, E) Tumor stage. (F) Positive Her-2 status. (G) Positive ER status. (H) Positive PR status. (I) Triple-negative breast cancer. (J, K) TNM stage. (L, M) Cluster state. (N, O) Age at diagnosis. Figure S6: the Kaplan–Meier curves show the six FRGs in the GSE21653 cohort. (A) CARS1, (B) CHAC1, (C) FANCD2, (D) AIFM2, (E) G6PD, and (F) HMOX1. Table S1: relationships between the expression of CARS1 and important clinical characteristics. Table S2: relationships between the expression of CHAC1 and important clinical characteristics. Table S3: relationships between the expression of FANCD2 and important clinical characteristics. Table S4: relationships between the expression of AIFM2 and important clinical characteristics. Table S5: relationships between the expression of G6PD and important clinical characteristics. Table S6: relationships between the expression of HMOX1 and important clinical characteristics. [file 6871518.f1.zip › Table S4.docx]

Table S4. Relationships between the expression of AIFM2 and important clinical characteristics.

| Characteristic | Low expression of AIFM2 | High expression of AIFM2 | p |
| --- | --- | --- | --- |
| T stage, n (%) |  |  | 0.049 |
| T1 | 131 (12.1%) | 146 (13.5%) |  |
| T2 | 318 (29.4%) | 311 (28.8%) |  |
| T3 | 67 (6.2%) | 72 (6.7%) |  |
| T4 | 25 (2.3%) | 10 (0.9%) |  |
| N stage, n (%) |  |  | 0.972 |
| N0 | 257 (24.2%) | 257 (24.2%) |  |
| N1 | 182 (17.1%) | 176 (16.5%) |  |
| N2 | 56 (5.3%) | 60 (5.6%) |  |
| N3 | 38 (3.6%) | 38 (3.6%) |  |
| M stage, n (%) |  |  | 1.000 |
| M0 | 453 (49.1%) | 449 (48.7%) |  |
| M1 | 10 (1.1%) | 10 (1.1%) |  |
| Pathologic stage, n (%) |  |  | 0.959 |
| Stage I | 89 (8.4%) | 92 (8.7%) |  |
| Stage II | 311 (29.3%) | 308 (29.1%) |  |
| Stage III | 120 (11.3%) | 122 (11.5%) |  |
| Stage IV | 10 (0.9%) | 8 (0.8%) |  |
| PR status, n (%) |  |  | 0.903 |
| Negative | 169 (16.3%) | 173 (16.7%) |  |
| Indeterminate | 2 (0.2%) | 2 (0.2%) |  |
| Positive | 349 (33.8%) | 339 (32.8%) |  |
| ER status, n (%) |  |  | 0.884 |
| Negative | 123 (11.9%) | 117 (11.3%) |  |
| Indeterminate | 1 (0.1%) | 1 (0.1%) |  |
| Positive | 396 (38.3%) | 397 (38.4%) |  |
| HER2 status, n (%) |  |  | 0.317 |
| Negative | 271 (37.3%) | 287 (39.5%) |  |
| Indeterminate | 6 (0.8%) | 6 (0.8%) |  |
| Positive | 87 (12%) | 70 (9.6%) |  |
| Molecular subtype, n (%) |  |  | 0.518 |
| Others | 21 (1.9%) | 19 (1.8%) |  |
| LumA | 287 (26.5%) | 275 (25.4%) |  |
| LumB | 94 (8.7%) | 110 (10.2%) |  |
| Her2 | 46 (4.2%) | 36 (3.3%) |  |
| Triple negative | 93 (8.6%) | 102 (9.4%) |  |
| Menopause status, n (%) |  |  | 0.965 |
| Pre | 116 (11.9%) | 113 (11.6%) |  |
| Peri | 20 (2.1%) | 20 (2.1%) |  |
| Post | 349 (35.9%) | 354 (36.4%) |  |
| Tumor location, n (%) |  |  | 0.604 |
| Left | 286 (26.4%) | 277 (25.6%) |  |
| Right | 255 (23.5%) | 265 (24.5%) |  |
