# Supplementary material for: Correlation between Ferroptosis-Related Gene Signature and Immune Landscape, Prognosis in Breast Cancer
Source: J Immunol Res. 2022 Oct 11;2022:6871518. doi: 10.1155/2022/6871518 (PMC9613394; doi:10.1155/2022/6871518)
Supplement: Supplementary Materials — Figure S1: identification of differentially expressed mRNAs between clusters 1 and 2 in the TCGA-BRCA cohort. (A) Volcano plot. (B) Heat map. Figure S2: the Gene Ontology annotation of differentially expressed genes. GO enrichment: (A) BP, (B) CC, and (C) MF. (D) KEGG enrichment. Figure S3: the Kaplan–Meier curves show the six FRGs in the TCGA-BRCA training cohort. (A) CARS1, (B) CHAC1, (C) FANCD2, (D) AIFM2, (E) G6PD, and (F) HMOX1. Figure S4: construction of a six-gene signature model in the TCGA-BRCA training cohort. (A) LASSO coefficient profiles of the expressions of the candidate genes. (B) Selection of the penalty parameter (λ) in the LASSO model via sixfold cross-validation. Figure S5: stratified analysis in the whole TCGA-BRCA set. (A, B) Lymph node metastasis. (C) Distant metastasis at diagnosis. (D, E) Tumor stage. (F) Positive Her-2 status. (G) Positive ER status. (H) Positive PR status. (I) Triple-negative breast cancer. (J, K) TNM stage. (L, M) Cluster state. (N, O) Age at diagnosis. Figure S6: the Kaplan–Meier curves show the six FRGs in the GSE21653 cohort. (A) CARS1, (B) CHAC1, (C) FANCD2, (D) AIFM2, (E) G6PD, and (F) HMOX1. Table S1: relationships between the expression of CARS1 and important clinical characteristics. Table S2: relationships between the expression of CHAC1 and important clinical characteristics. Table S3: relationships between the expression of FANCD2 and important clinical characteristics. Table S4: relationships between the expression of AIFM2 and important clinical characteristics. Table S5: relationships between the expression of G6PD and important clinical characteristics. Table S6: relationships between the expression of HMOX1 and important clinical characteristics. [file 6871518.f1.zip › Table S5.docx]

Table S5. Relationships between the expression of G6PD and important clinical characteristics.

| Characteristic | Low expression of G6PD | High expression of G6PD | p |
| --- | --- | --- | --- |
| T stage, n (%) |  |  | 0.054 |
| T1 | 158 (14.6%) | 119 (11%) |  |
| T2 | 303 (28.1%) | 326 (30.2%) |  |
| T3 | 65 (6%) | 74 (6.9%) |  |
| T4 | 15 (1.4%) | 20 (1.9%) |  |
| N stage, n (%) |  |  | 0.091 |
| N0 | 276 (25.9%) | 238 (22.4%) |  |
| N1 | 176 (16.5%) | 182 (17.1%) |  |
| N2 | 51 (4.8%) | 65 (6.1%) |  |
| N3 | 32 (3%) | 44 (4.1%) |  |
| M stage, n (%) |  |  | 0.146 |
| M0 | 442 (47.9%) | 460 (49.9%) |  |
| M1 | 6 (0.7%) | 14 (1.5%) |  |
| Pathologic stage, n (%) |  |  | 0.003 |
| Stage I | 109 (10.3%) | 72 (6.8%) |  |
| Stage II | 308 (29.1%) | 311 (29.3%) |  |
| Stage III | 108 (10.2%) | 134 (12.6%) |  |
| Stage IV | 5 (0.5%) | 13 (1.2%) |  |
| PR status, n (%) |  |  | 0.216 |
| Negative | 160 (15.5%) | 182 (17.6%) |  |
| Indeterminate | 3 (0.3%) | 1 (0.1%) |  |
| Positive | 357 (34.5%) | 331 (32%) |  |
| ER status, n (%) |  |  | 0.108 |
| Negative | 108 (10.4%) | 132 (12.8%) |  |
| Indeterminate | 1 (0.1%) | 1 (0.1%) |  |
| Positive | 411 (39.7%) | 382 (36.9%) |  |
| HER2 status, n (%) |  |  | 0.010 |
| Negative | 288 (39.6%) | 270 (37.1%) |  |
| Indeterminate | 7 (1%) | 5 (0.7%) |  |
| Positive | 60 (8.3%) | 97 (13.3%) |  |
| Molecular subtype, n (%) |  |  | < 0.001 |
| Others | 22 (2%) | 18 (1.7%) |  |
| LumA | 322 (29.7%) | 240 (22.2%) |  |
| LumB | 80 (7.4%) | 124 (11.4%) |  |
| Her2 | 7 (0.6%) | 75 (6.9%) |  |
| Triple negative | 110 (10.2%) | 85 (7.8%) |  |
| Menopause status, n (%) |  |  | 0.830 |
| Pre | 112 (11.5%) | 117 (12%) |  |
| Peri | 20 (2.1%) | 20 (2.1%) |  |
| Post | 360 (37%) | 343 (35.3%) |  |
| Tumor location, n (%) |  |  | 0.073 |
| Left | 266 (24.6%) | 297 (27.4%) |  |
| Right | 275 (25.4%) | 245 (22.6%) |  |
