# Supplementary material for: Correlation between Ferroptosis-Related Gene Signature and Immune Landscape, Prognosis in Breast Cancer
Source: J Immunol Res. 2022 Oct 11;2022:6871518. doi: 10.1155/2022/6871518 (PMC9613394; doi:10.1155/2022/6871518)
Supplement: Supplementary Materials — Figure S1: identification of differentially expressed mRNAs between clusters 1 and 2 in the TCGA-BRCA cohort. (A) Volcano plot. (B) Heat map. Figure S2: the Gene Ontology annotation of differentially expressed genes. GO enrichment: (A) BP, (B) CC, and (C) MF. (D) KEGG enrichment. Figure S3: the Kaplan–Meier curves show the six FRGs in the TCGA-BRCA training cohort. (A) CARS1, (B) CHAC1, (C) FANCD2, (D) AIFM2, (E) G6PD, and (F) HMOX1. Figure S4: construction of a six-gene signature model in the TCGA-BRCA training cohort. (A) LASSO coefficient profiles of the expressions of the candidate genes. (B) Selection of the penalty parameter (λ) in the LASSO model via sixfold cross-validation. Figure S5: stratified analysis in the whole TCGA-BRCA set. (A, B) Lymph node metastasis. (C) Distant metastasis at diagnosis. (D, E) Tumor stage. (F) Positive Her-2 status. (G) Positive ER status. (H) Positive PR status. (I) Triple-negative breast cancer. (J, K) TNM stage. (L, M) Cluster state. (N, O) Age at diagnosis. Figure S6: the Kaplan–Meier curves show the six FRGs in the GSE21653 cohort. (A) CARS1, (B) CHAC1, (C) FANCD2, (D) AIFM2, (E) G6PD, and (F) HMOX1. Table S1: relationships between the expression of CARS1 and important clinical characteristics. Table S2: relationships between the expression of CHAC1 and important clinical characteristics. Table S3: relationships between the expression of FANCD2 and important clinical characteristics. Table S4: relationships between the expression of AIFM2 and important clinical characteristics. Table S5: relationships between the expression of G6PD and important clinical characteristics. Table S6: relationships between the expression of HMOX1 and important clinical characteristics. [file 6871518.f1.zip › Table S6.docx]

Table S6. Relationships between the expression of HMOX1 and important clinical characteristics.

| Characteristic | Low expression of HMOX1 | High expression of HMOX1 | p |
| --- | --- | --- | --- |
| T stage, n (%) |  |  | 0.370 |
| T1 | 136 (12.6%) | 141 (13.1%) |  |
| T2 | 312 (28.9%) | 317 (29.4%) |  |
| T3 | 77 (7.1%) | 62 (5.7%) |  |
| T4 | 14 (1.3%) | 21 (1.9%) |  |
| N stage, n (%) |  |  | 0.098 |
| N0 | 262 (24.6%) | 252 (23.7%) |  |
| N1 | 190 (17.9%) | 168 (15.8%) |  |
| N2 | 48 (4.5%) | 68 (6.4%) |  |
| N3 | 33 (3.1%) | 43 (4%) |  |
| M stage, n (%) |  |  | 0.852 |
| M0 | 454 (49.2%) | 448 (48.6%) |  |
| M1 | 11 (1.2%) | 9 (1%) |  |
| Pathologic stage, n (%) |  |  | 0.512 |
| Stage I | 96 (9.1%) | 85 (8%) |  |
| Stage II | 313 (29.5%) | 306 (28.9%) |  |
| Stage III | 112 (10.6%) | 130 (12.3%) |  |
| Stage IV | 10 (0.9%) | 8 (0.8%) |  |
| PR status, n (%) |  |  | 0.498 |
| Negative | 162 (15.7%) | 180 (17.4%) |  |
| Indeterminate | 2 (0.2%) | 2 (0.2%) |  |
| Positive | 351 (33.9%) | 337 (32.6%) |  |
| ER status, n (%) |  |  | 0.093 |
| Negative | 109 (10.5%) | 131 (12.7%) |  |
| Indeterminate | 2 (0.2%) | 0 (0%) |  |
| Positive | 405 (39.1%) | 388 (37.5%) |  |
| HER2 status, n (%) |  |  | 0.729 |
| Negative | 264 (36.3%) | 294 (40.4%) |  |
| Indeterminate | 5 (0.7%) | 7 (1%) |  |
| Positive | 79 (10.9%) | 78 (10.7%) |  |
| Molecular subtype, n (%) |  |  | 0.002 |
| Others | 21 (1.9%) | 19 (1.8%) |  |
| LumA | 313 (28.9%) | 249 (23%) |  |
| LumB | 85 (7.8%) | 119 (11%) |  |
| Her2 | 34 (3.1%) | 48 (4.4%) |  |
| Triple negative | 88 (8.1%) | 107 (9.9%) |  |
| Menopause status, n (%) |  |  | 0.015 |
| Pre | 130 (13.4%) | 99 (10.2%) |  |
| Peri | 23 (2.4%) | 17 (1.7%) |  |
| Post | 327 (33.6%) | 376 (38.7%) |  |
| Tumor location, n (%) |  |  | 0.346 |
| Left | 273 (25.2%) | 290 (26.8%) |  |
| Right | 268 (24.7%) | 252 (23.3%) |  |
