# Supplementary figures and images for: Correlation between Ferroptosis-Related Gene Signature and Immune Landscape, Prognosis in Breast Cancer
Source: J Immunol Res. 2022 Oct 11;2022:6871518. doi: 10.1155/2022/6871518 (PMC9613394; doi:10.1155/2022/6871518)

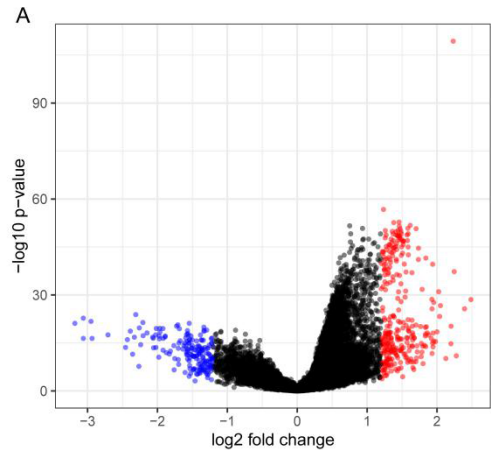

change

- down-regulated
- not-changed
- up-regulated

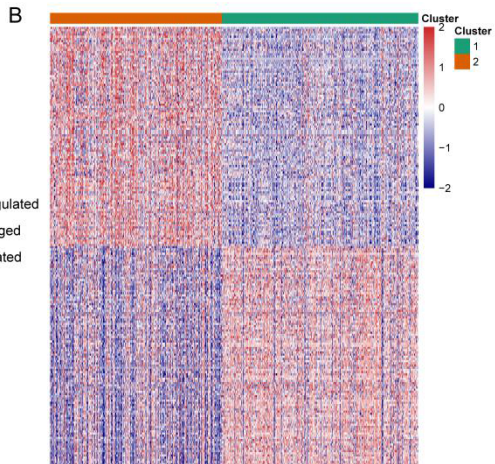

Supplement: Supplementary Materials — Figure S1: identification of differentially expressed mRNAs between clusters 1 and 2 in the TCGA-BRCA cohort. (A) Volcano plot. (B) Heat map. Figure S2: the Gene Ontology annotation of differentially expressed genes. GO enrichment: (A) BP, (B) CC, and (C) MF. (D) KEGG enrichment. Figure S3: the Kaplan–Meier curves show the six FRGs in the TCGA-BRCA training cohort. (A) CARS1, (B) CHAC1, (C) FANCD2, (D) AIFM2, (E) G6PD, and (F) HMOX1. Figure S4: construction of a six-gene signature model in the TCGA-BRCA training cohort. (A) LASSO coefficient profiles of the expressions of the candidate genes. (B) Selection of the penalty parameter (λ) in the LASSO model via sixfold cross-validation. Figure S5: stratified analysis in the whole TCGA-BRCA set. (A, B) Lymph node metastasis. (C) Distant metastasis at diagnosis. (D, E) Tumor stage. (F) Positive Her-2 status. (G) Positive ER status. (H) Positive PR status. (I) Triple-negative breast cancer. (J, K) TNM stage. (L, M) Cluster state. (N, O) Age at diagnosis. Figure S6: the Kaplan–Meier curves show the six FRGs in the GSE21653 cohort. (A) CARS1, (B) CHAC1, (C) FANCD2, (D) AIFM2, (E) G6PD, and (F) HMOX1. Table S1: relationships between the expression of CARS1 and important clinical characteristics. Table S2: relationships between the expression of CHAC1 and important clinical characteristics. Table S3: relationships between the expression of FANCD2 and important clinical characteristics. Table S4: relationships between the expression of AIFM2 and important clinical characteristics. Table S5: relationships between the expression of G6PD and important clinical characteristics. Table S6: relationships between the expression of HMOX1 and important clinical characteristics. [file 6871518.f1.zip › Figure S1.pdf]

A

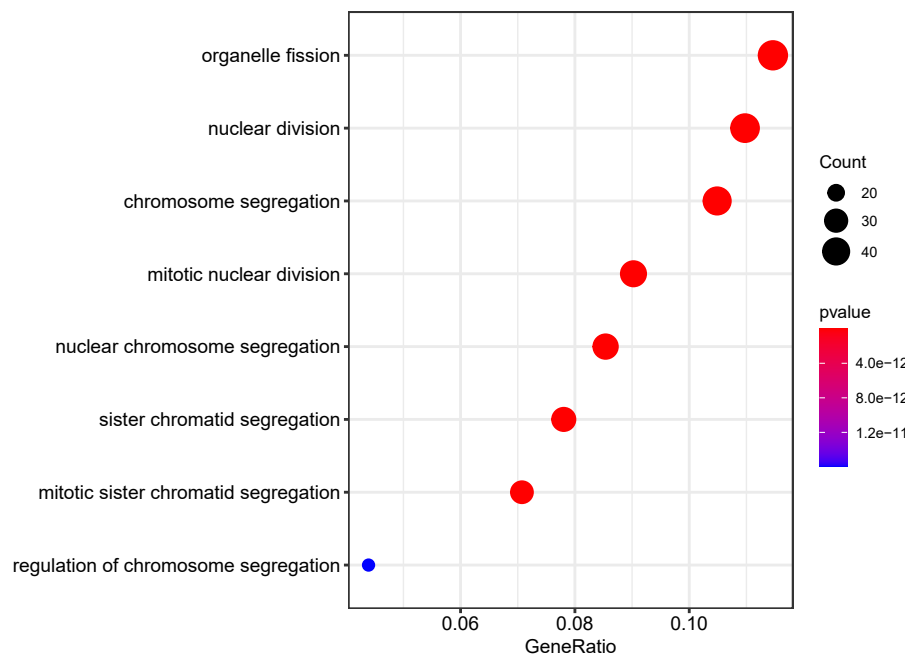

B

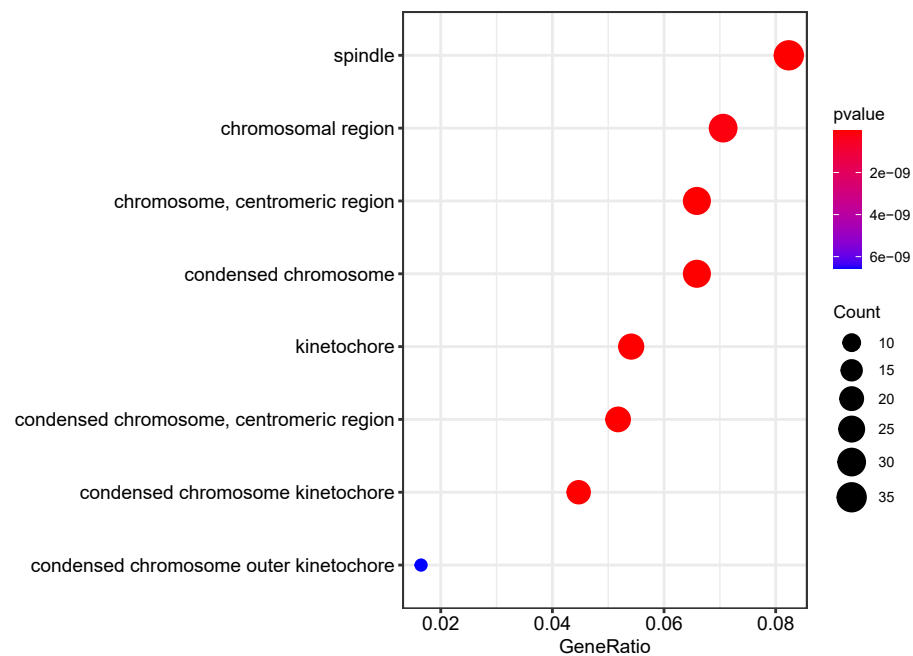

C

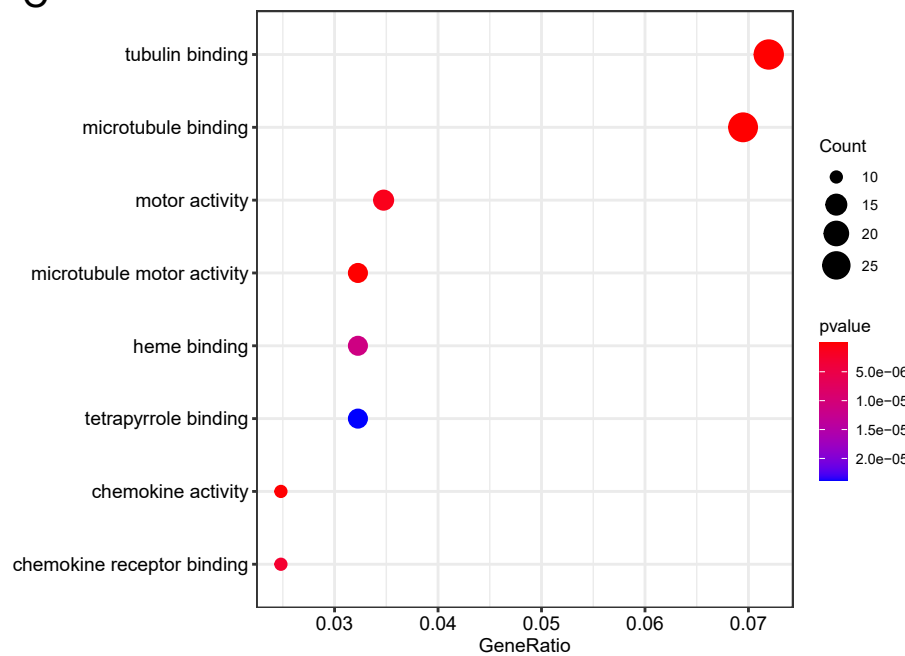

D

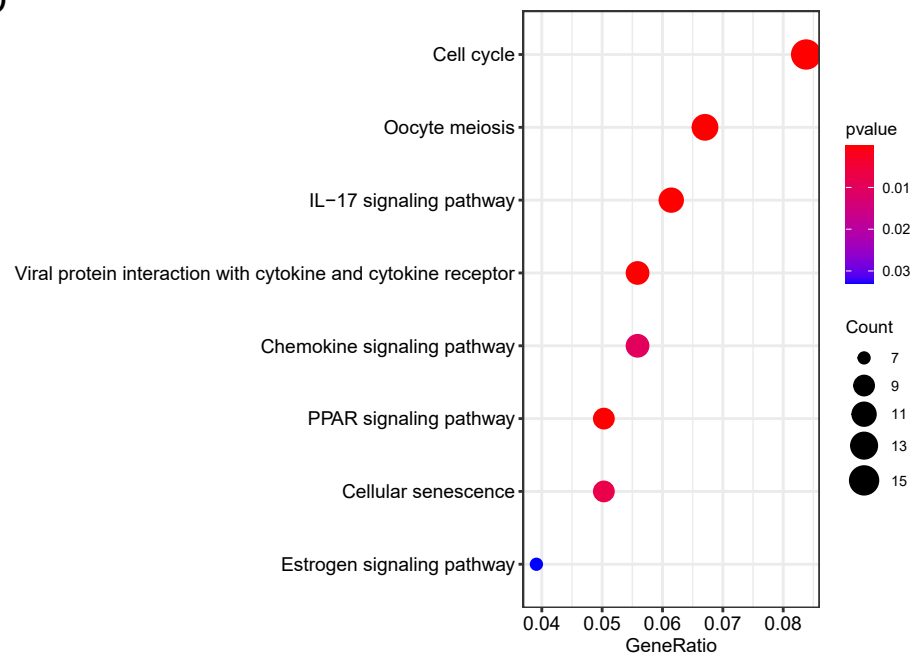

Supplement: Supplementary Materials — Figure S1: identification of differentially expressed mRNAs between clusters 1 and 2 in the TCGA-BRCA cohort. (A) Volcano plot. (B) Heat map. Figure S2: the Gene Ontology annotation of differentially expressed genes. GO enrichment: (A) BP, (B) CC, and (C) MF. (D) KEGG enrichment. Figure S3: the Kaplan–Meier curves show the six FRGs in the TCGA-BRCA training cohort. (A) CARS1, (B) CHAC1, (C) FANCD2, (D) AIFM2, (E) G6PD, and (F) HMOX1. Figure S4: construction of a six-gene signature model in the TCGA-BRCA training cohort. (A) LASSO coefficient profiles of the expressions of the candidate genes. (B) Selection of the penalty parameter (λ) in the LASSO model via sixfold cross-validation. Figure S5: stratified analysis in the whole TCGA-BRCA set. (A, B) Lymph node metastasis. (C) Distant metastasis at diagnosis. (D, E) Tumor stage. (F) Positive Her-2 status. (G) Positive ER status. (H) Positive PR status. (I) Triple-negative breast cancer. (J, K) TNM stage. (L, M) Cluster state. (N, O) Age at diagnosis. Figure S6: the Kaplan–Meier curves show the six FRGs in the GSE21653 cohort. (A) CARS1, (B) CHAC1, (C) FANCD2, (D) AIFM2, (E) G6PD, and (F) HMOX1. Table S1: relationships between the expression of CARS1 and important clinical characteristics. Table S2: relationships between the expression of CHAC1 and important clinical characteristics. Table S3: relationships between the expression of FANCD2 and important clinical characteristics. Table S4: relationships between the expression of AIFM2 and important clinical characteristics. Table S5: relationships between the expression of G6PD and important clinical characteristics. Table S6: relationships between the expression of HMOX1 and important clinical characteristics. [file 6871518.f1.zip › Figure S2.pdf]

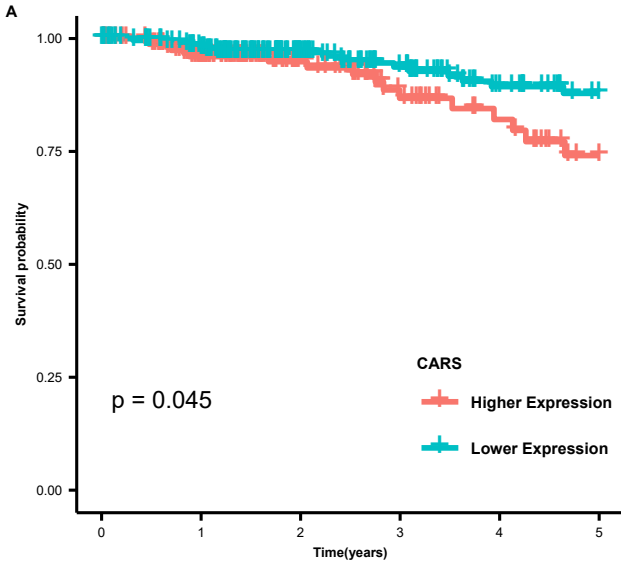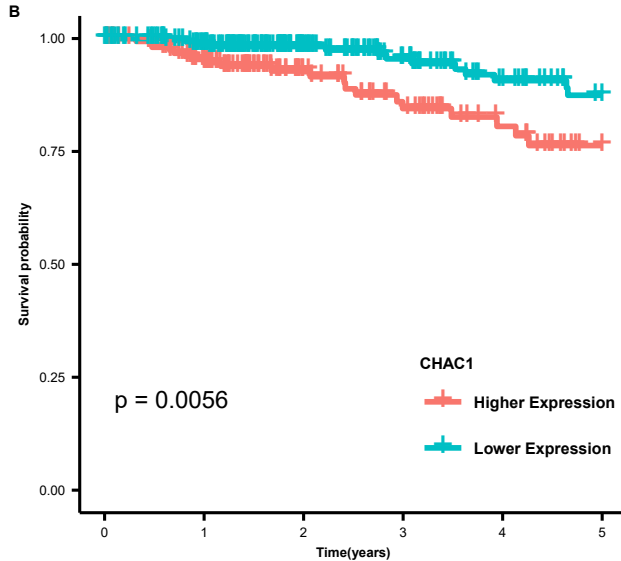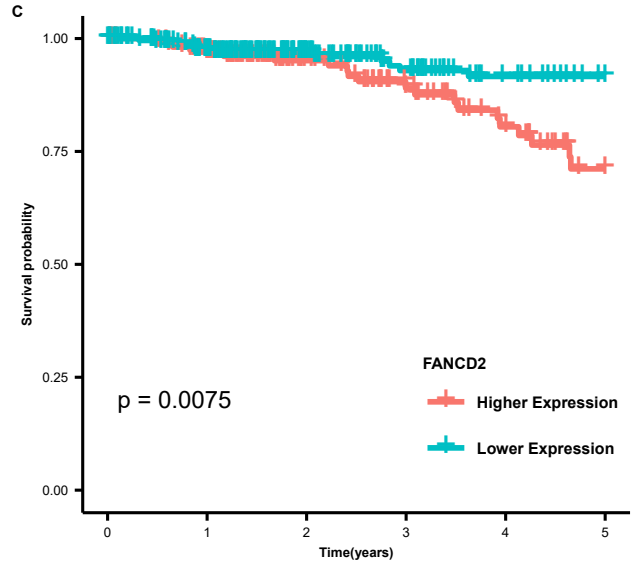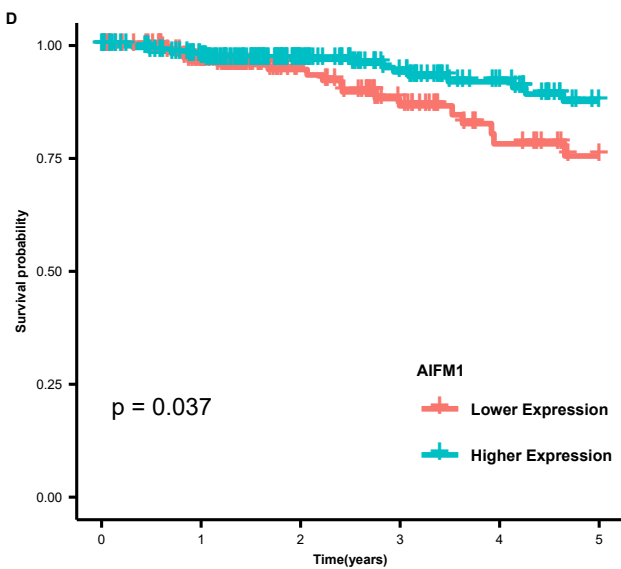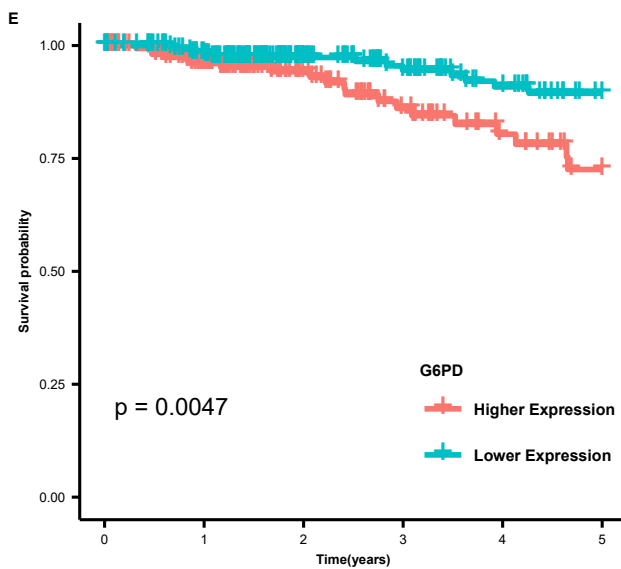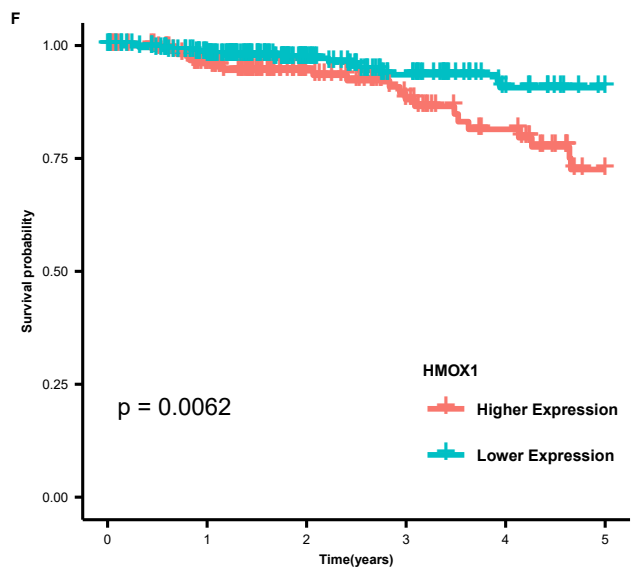

Supplement: Supplementary Materials — Figure S1: identification of differentially expressed mRNAs between clusters 1 and 2 in the TCGA-BRCA cohort. (A) Volcano plot. (B) Heat map. Figure S2: the Gene Ontology annotation of differentially expressed genes. GO enrichment: (A) BP, (B) CC, and (C) MF. (D) KEGG enrichment. Figure S3: the Kaplan–Meier curves show the six FRGs in the TCGA-BRCA training cohort. (A) CARS1, (B) CHAC1, (C) FANCD2, (D) AIFM2, (E) G6PD, and (F) HMOX1. Figure S4: construction of a six-gene signature model in the TCGA-BRCA training cohort. (A) LASSO coefficient profiles of the expressions of the candidate genes. (B) Selection of the penalty parameter (λ) in the LASSO model via sixfold cross-validation. Figure S5: stratified analysis in the whole TCGA-BRCA set. (A, B) Lymph node metastasis. (C) Distant metastasis at diagnosis. (D, E) Tumor stage. (F) Positive Her-2 status. (G) Positive ER status. (H) Positive PR status. (I) Triple-negative breast cancer. (J, K) TNM stage. (L, M) Cluster state. (N, O) Age at diagnosis. Figure S6: the Kaplan–Meier curves show the six FRGs in the GSE21653 cohort. (A) CARS1, (B) CHAC1, (C) FANCD2, (D) AIFM2, (E) G6PD, and (F) HMOX1. Table S1: relationships between the expression of CARS1 and important clinical characteristics. Table S2: relationships between the expression of CHAC1 and important clinical characteristics. Table S3: relationships between the expression of FANCD2 and important clinical characteristics. Table S4: relationships between the expression of AIFM2 and important clinical characteristics. Table S5: relationships between the expression of G6PD and important clinical characteristics. Table S6: relationships between the expression of HMOX1 and important clinical characteristics. [file 6871518.f1.zip › Figure S3.pdf]

A

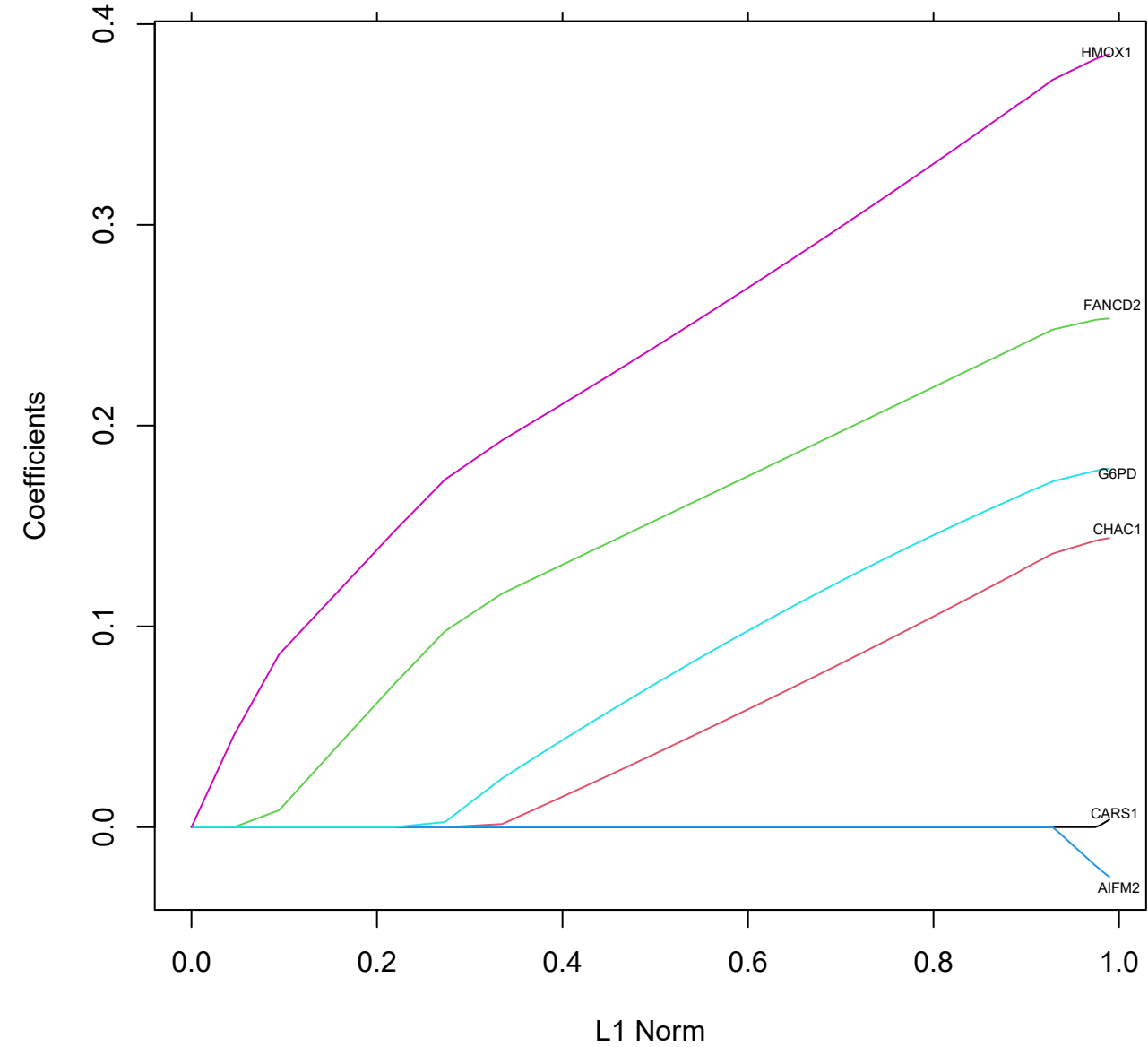

B

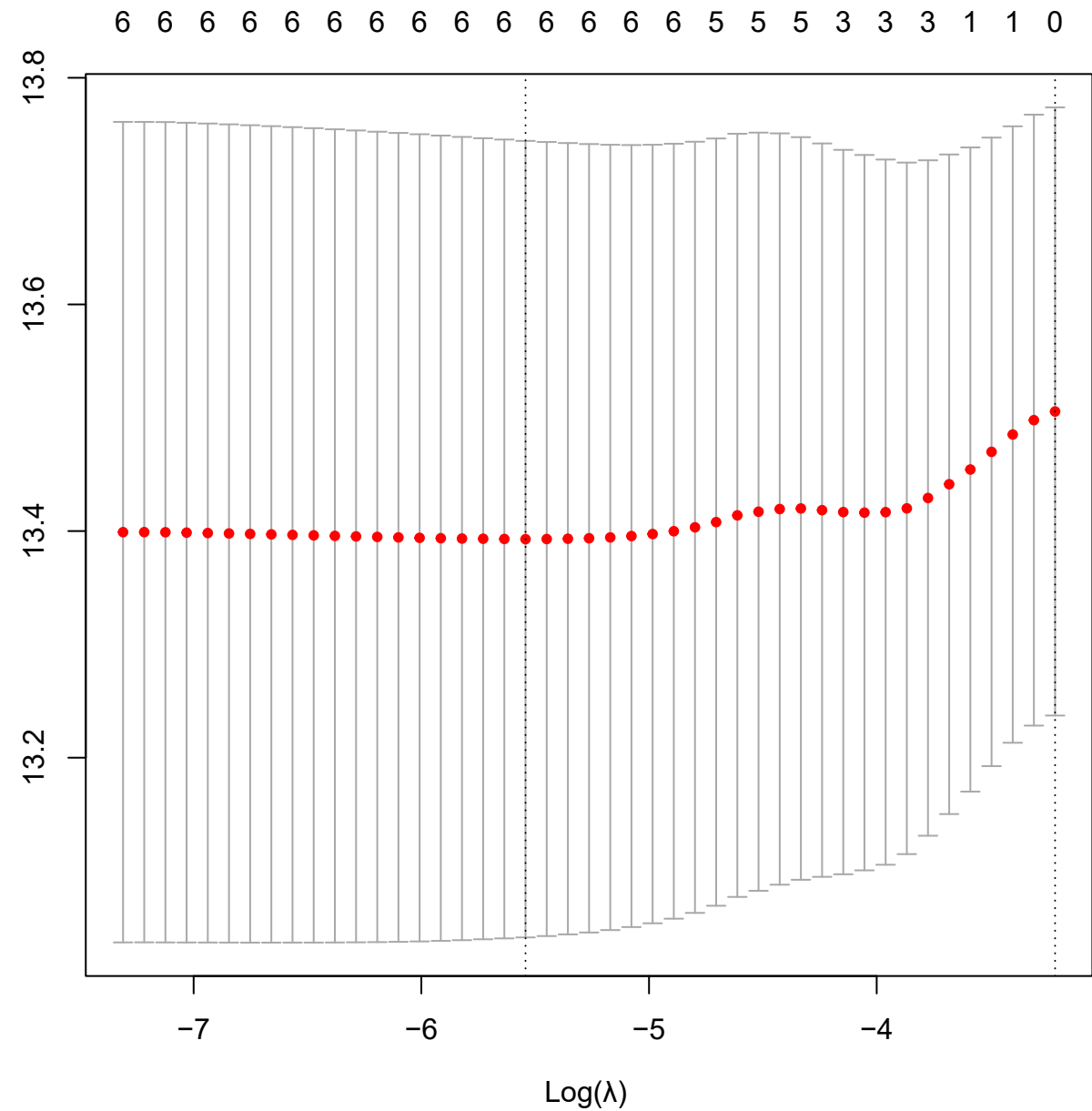

Supplement: Supplementary Materials — Figure S1: identification of differentially expressed mRNAs between clusters 1 and 2 in the TCGA-BRCA cohort. (A) Volcano plot. (B) Heat map. Figure S2: the Gene Ontology annotation of differentially expressed genes. GO enrichment: (A) BP, (B) CC, and (C) MF. (D) KEGG enrichment. Figure S3: the Kaplan–Meier curves show the six FRGs in the TCGA-BRCA training cohort. (A) CARS1, (B) CHAC1, (C) FANCD2, (D) AIFM2, (E) G6PD, and (F) HMOX1. Figure S4: construction of a six-gene signature model in the TCGA-BRCA training cohort. (A) LASSO coefficient profiles of the expressions of the candidate genes. (B) Selection of the penalty parameter (λ) in the LASSO model via sixfold cross-validation. Figure S5: stratified analysis in the whole TCGA-BRCA set. (A, B) Lymph node metastasis. (C) Distant metastasis at diagnosis. (D, E) Tumor stage. (F) Positive Her-2 status. (G) Positive ER status. (H) Positive PR status. (I) Triple-negative breast cancer. (J, K) TNM stage. (L, M) Cluster state. (N, O) Age at diagnosis. Figure S6: the Kaplan–Meier curves show the six FRGs in the GSE21653 cohort. (A) CARS1, (B) CHAC1, (C) FANCD2, (D) AIFM2, (E) G6PD, and (F) HMOX1. Table S1: relationships between the expression of CARS1 and important clinical characteristics. Table S2: relationships between the expression of CHAC1 and important clinical characteristics. Table S3: relationships between the expression of FANCD2 and important clinical characteristics. Table S4: relationships between the expression of AIFM2 and important clinical characteristics. Table S5: relationships between the expression of G6PD and important clinical characteristics. Table S6: relationships between the expression of HMOX1 and important clinical characteristics. [file 6871518.f1.zip › Figure S4.pdf]

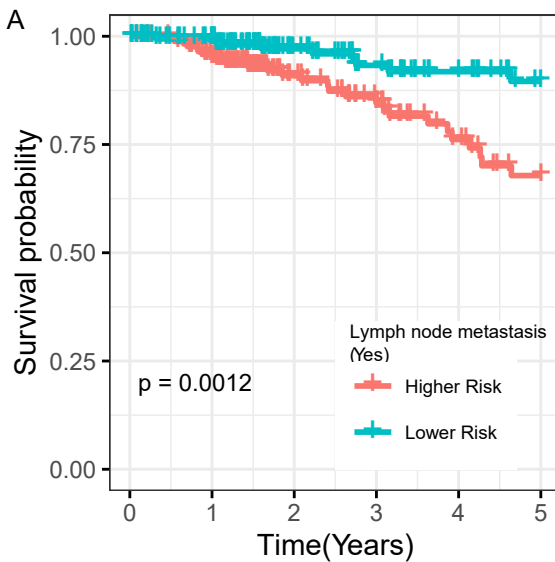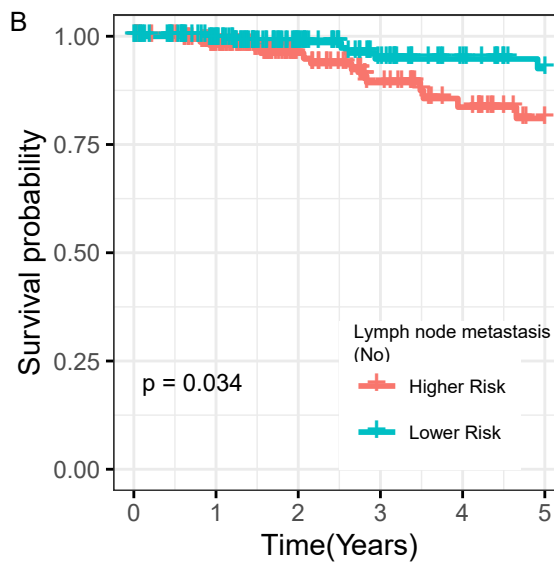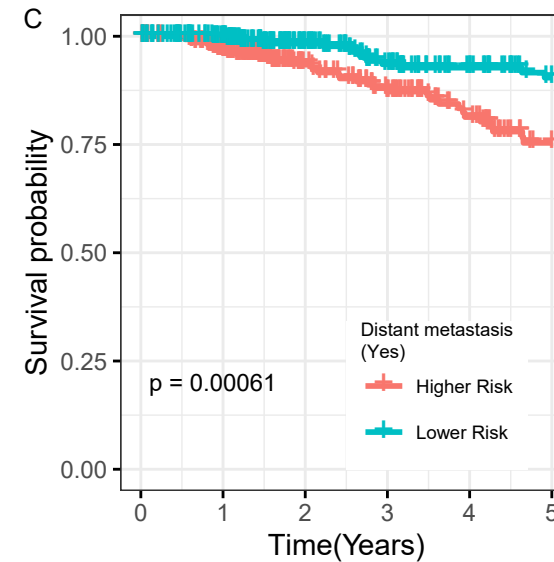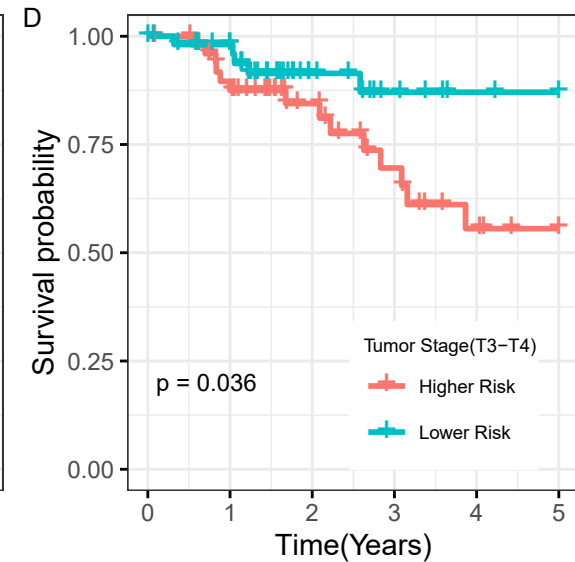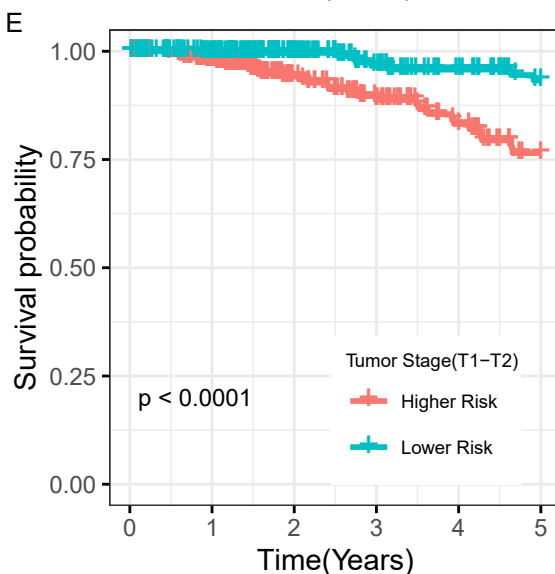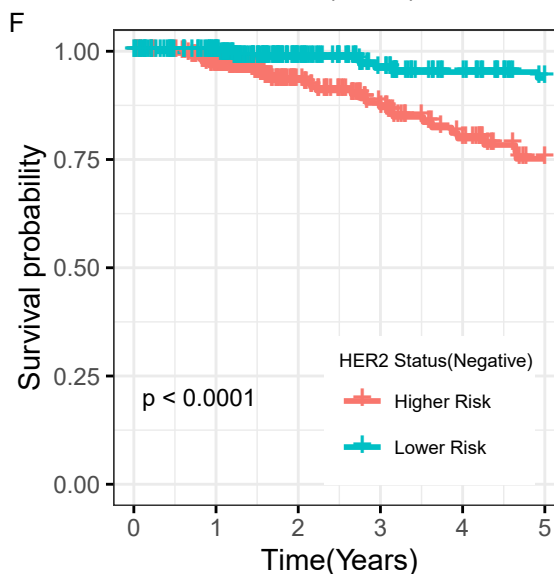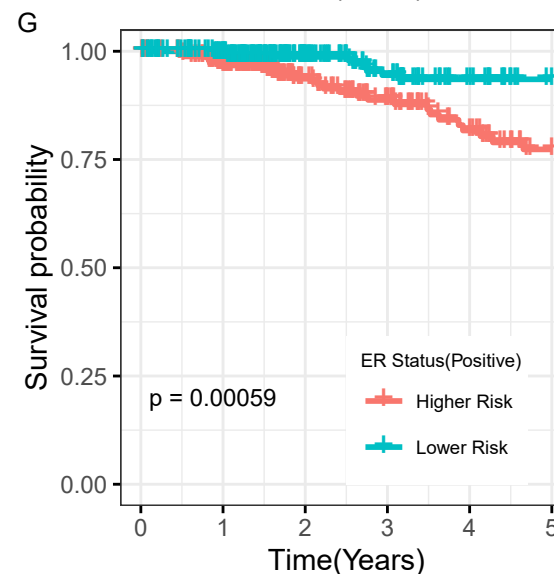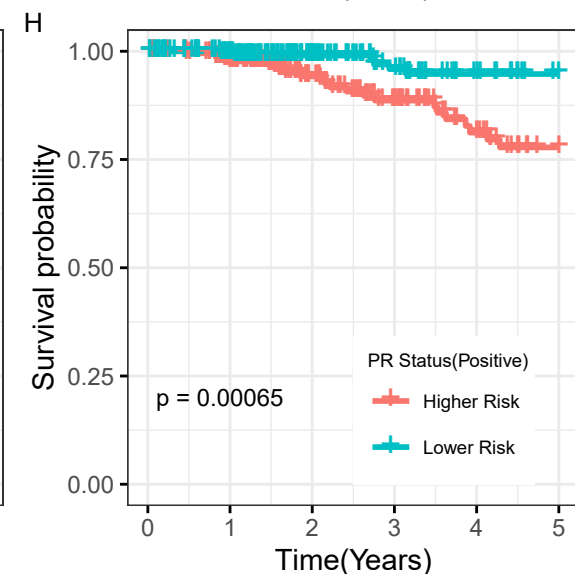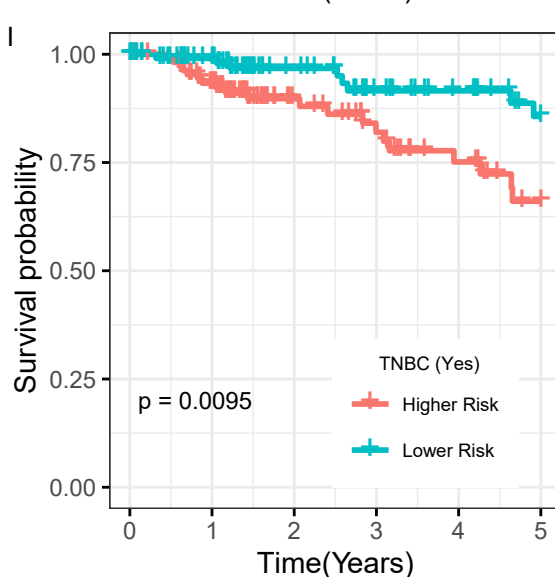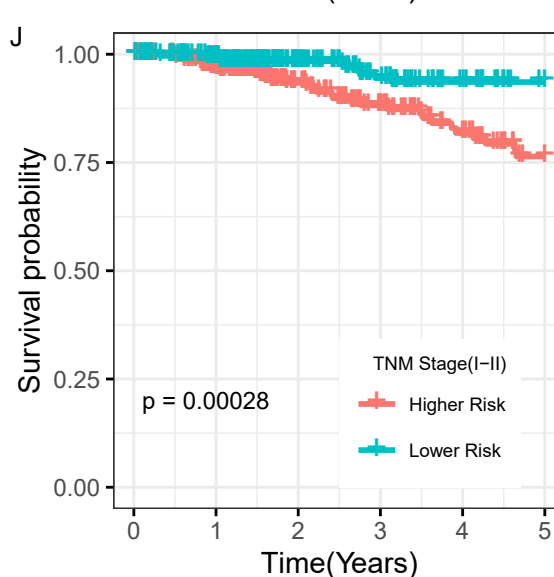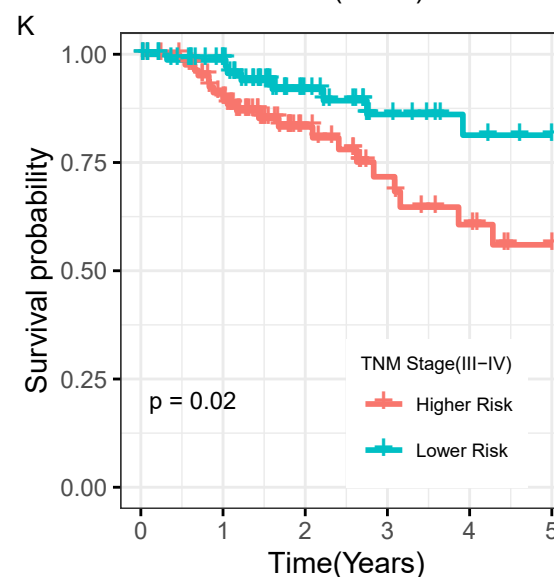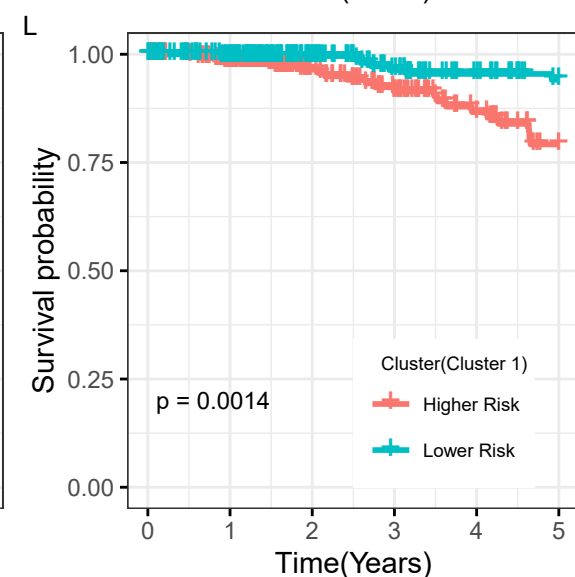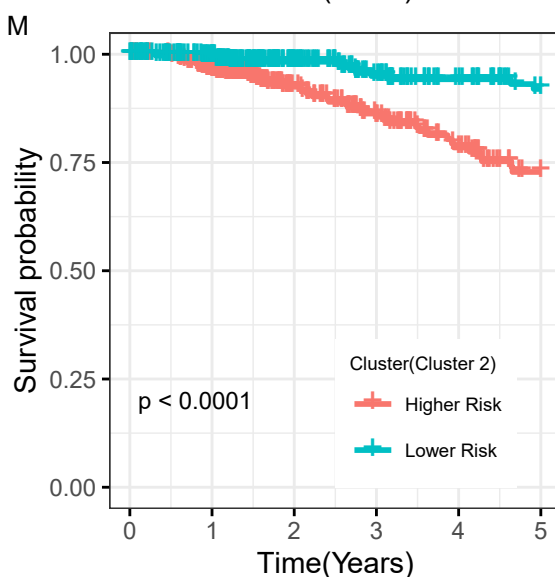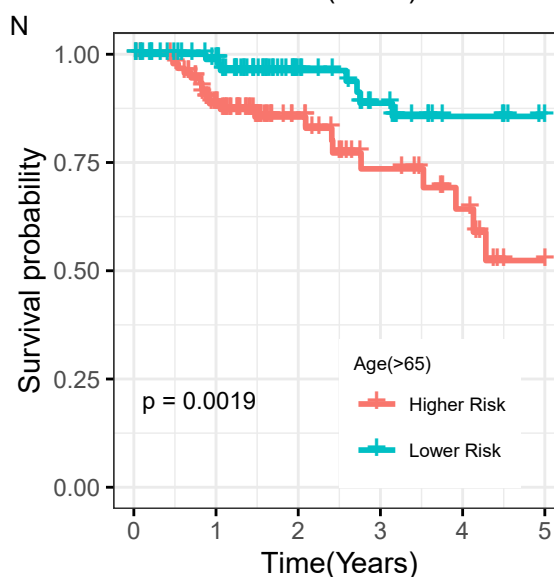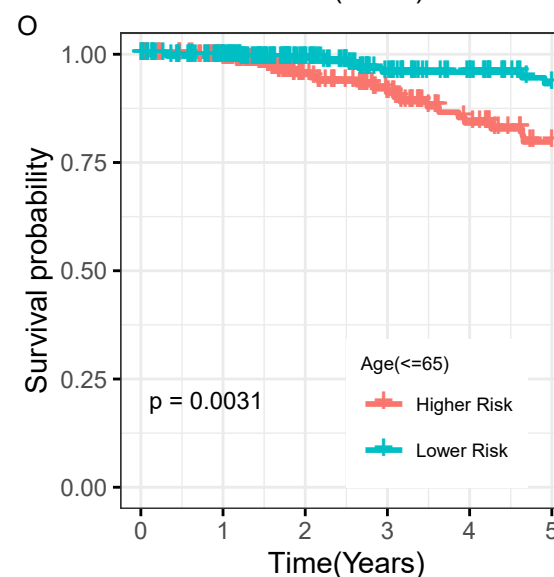

Supplement: Supplementary Materials — Figure S1: identification of differentially expressed mRNAs between clusters 1 and 2 in the TCGA-BRCA cohort. (A) Volcano plot. (B) Heat map. Figure S2: the Gene Ontology annotation of differentially expressed genes. GO enrichment: (A) BP, (B) CC, and (C) MF. (D) KEGG enrichment. Figure S3: the Kaplan–Meier curves show the six FRGs in the TCGA-BRCA training cohort. (A) CARS1, (B) CHAC1, (C) FANCD2, (D) AIFM2, (E) G6PD, and (F) HMOX1. Figure S4: construction of a six-gene signature model in the TCGA-BRCA training cohort. (A) LASSO coefficient profiles of the expressions of the candidate genes. (B) Selection of the penalty parameter (λ) in the LASSO model via sixfold cross-validation. Figure S5: stratified analysis in the whole TCGA-BRCA set. (A, B) Lymph node metastasis. (C) Distant metastasis at diagnosis. (D, E) Tumor stage. (F) Positive Her-2 status. (G) Positive ER status. (H) Positive PR status. (I) Triple-negative breast cancer. (J, K) TNM stage. (L, M) Cluster state. (N, O) Age at diagnosis. Figure S6: the Kaplan–Meier curves show the six FRGs in the GSE21653 cohort. (A) CARS1, (B) CHAC1, (C) FANCD2, (D) AIFM2, (E) G6PD, and (F) HMOX1. Table S1: relationships between the expression of CARS1 and important clinical characteristics. Table S2: relationships between the expression of CHAC1 and important clinical characteristics. Table S3: relationships between the expression of FANCD2 and important clinical characteristics. Table S4: relationships between the expression of AIFM2 and important clinical characteristics. Table S5: relationships between the expression of G6PD and important clinical characteristics. Table S6: relationships between the expression of HMOX1 and important clinical characteristics. [file 6871518.f1.zip › Figure S5.pdf]

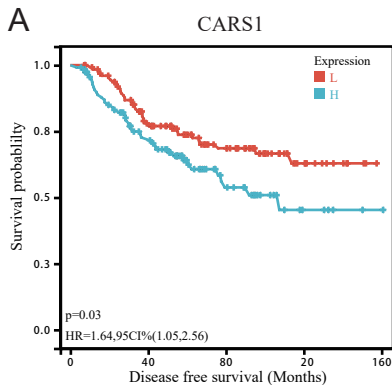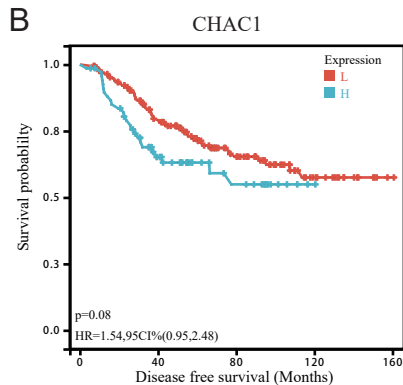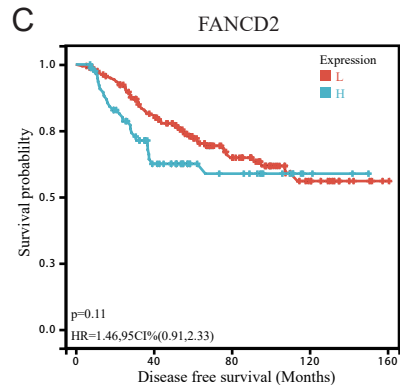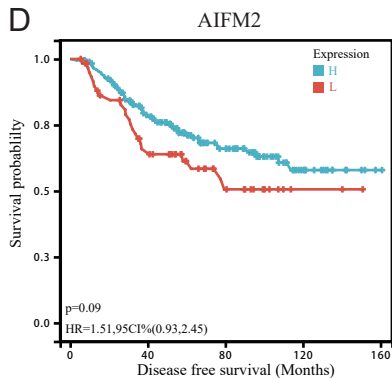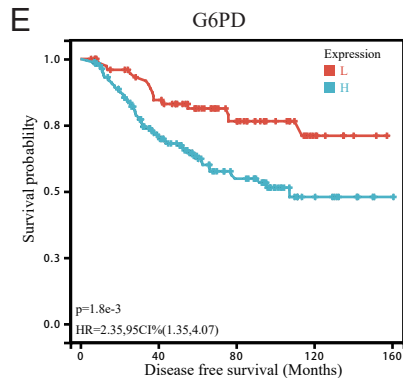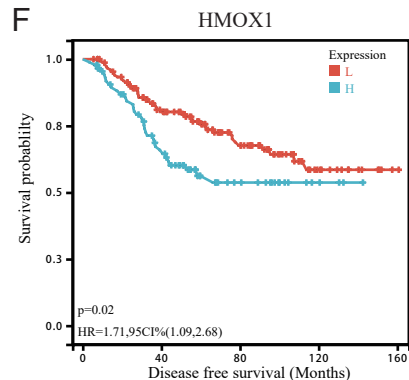

Supplement: Supplementary Materials — Figure S1: identification of differentially expressed mRNAs between clusters 1 and 2 in the TCGA-BRCA cohort. (A) Volcano plot. (B) Heat map. Figure S2: the Gene Ontology annotation of differentially expressed genes. GO enrichment: (A) BP, (B) CC, and (C) MF. (D) KEGG enrichment. Figure S3: the Kaplan–Meier curves show the six FRGs in the TCGA-BRCA training cohort. (A) CARS1, (B) CHAC1, (C) FANCD2, (D) AIFM2, (E) G6PD, and (F) HMOX1. Figure S4: construction of a six-gene signature model in the TCGA-BRCA training cohort. (A) LASSO coefficient profiles of the expressions of the candidate genes. (B) Selection of the penalty parameter (λ) in the LASSO model via sixfold cross-validation. Figure S5: stratified analysis in the whole TCGA-BRCA set. (A, B) Lymph node metastasis. (C) Distant metastasis at diagnosis. (D, E) Tumor stage. (F) Positive Her-2 status. (G) Positive ER status. (H) Positive PR status. (I) Triple-negative breast cancer. (J, K) TNM stage. (L, M) Cluster state. (N, O) Age at diagnosis. Figure S6: the Kaplan–Meier curves show the six FRGs in the GSE21653 cohort. (A) CARS1, (B) CHAC1, (C) FANCD2, (D) AIFM2, (E) G6PD, and (F) HMOX1. Table S1: relationships between the expression of CARS1 and important clinical characteristics. Table S2: relationships between the expression of CHAC1 and important clinical characteristics. Table S3: relationships between the expression of FANCD2 and important clinical characteristics. Table S4: relationships between the expression of AIFM2 and important clinical characteristics. Table S5: relationships between the expression of G6PD and important clinical characteristics. Table S6: relationships between the expression of HMOX1 and important clinical characteristics. [file 6871518.f1.zip › Figure S6.pdf]
